# Circulating fibroblasts and neutrophils co-expressing CDH11^+^ and chemokine receptors in rheumatoid and psoriatic arthritis: a shared mechanism of ‘arthritis spreading’?

**DOI:** 10.3389/fimmu.2025.1743919

**Published:** 2026-01-16

**Authors:** Maria Kyriakidi, Eleni-Kyriaki Vetsika, George E. Fragoulis, Maria Sakkou, Kleio-Maria Verrou, Anastasios Mourikis, Nikolaos I. Vlachogiannis, Maria G. Tektonidou, Petros P. Sfikakis

**Affiliations:** 1 Centre of New Biotechnologies and Precision Medicine (CNBPM), School of Medicine, National and Kapodistrian University of Athens, Athens, Greece; 2 Joint Rheumatology Program, School of Medicine, National and Kapodistrian University of Athens, Athens, Greece; 3 Department of Basic and Clinical Sciences, Medical School, University of Nicosia, UNIC Athens, Athens, Greece; 4 Third Department of Orthopaedics, KAT Attica General Hospital, Athens, Greece

**Keywords:** fibroblasts, fibrocytes, mass cytometry, neutrophils, psoriatic arthritis, rheumatoid arthritis

## Abstract

**Background:**

The mechanisms underlying the progression of chronic inflammatory arthritis remain largely unclear.

**Methods:**

We used single-cell mass cytometry on peripheral blood from patients with active rheumatoid arthritis (RA; n=11), psoriatic arthritis (PsA; n=12), and controls (n=9) to identify circulating cells co-expressing mesenchymal markers, including the homotypic adhesion molecule cadherin-11 (CDH11), and chemokine receptors. Proteomic profiling was performed on matched plasma using Olink. Confocal microscopy was used to investigate cell localization in synovial tissue.

**Results:**

Circulating cells co-expressing mesenchymal markers, including the adhesion molecule cadherin-11 (CDH11) and chemokine receptors, were identified. Of them, circulating fibroblasts (podoplanin^+^CD45^-^CD3^-^CD19^-^CD4^-^CD8^-^CD56^-^CD66b^-^CD294^-^) co-expressing CDH11 and C-C Chemokine Receptor 7 (CCR7) were found exclusively in arthritis patients’ blood. These cells were not detected in paired bone marrow samples, suggesting their potential origin from inflamed joints. Increased circulating fibrocytes (CD34^+^HLA-DR^+^CD45^+^CD3^-^CD19^-^CD4^-^CD8^-^CD56^-^CD66b^-^CD294^-^) co-expressing CDH11 and CCR7 were also found in patients’ blood. These cells were more prevalent in bone marrow, supporting their bone marrow origin. Among various leukocyte subsets, CDH11^+^CD90^+^CCR6^+^neutrophils were markedly elevated in both RA and PsA. These populations persisted after 3 months of antirheumatic drug administration, regardless of treatment response. Proteomic profiling on matched plasma using Olink, revealed that the presence of circulating CDH11^+^fibroblasts was associated with higher C-X-C Motif Chemokine Ligand 1 (CXCL1), Angiopoietin 1 (ANGPT1) and Tissue Inhibitor of Metalloproteinase 3 (TIMP3) levels, proteins involved in angiogenesis, chemotaxis, and matrix remodeling, respectively. Moreover, in patients with detectable circulating CDH11^+^fibroblasts chemokine signaling and cell-substrate adhesion were enriched. Finally, CDH11^+^neutrophils were identified in close proximity to synovial fibroblasts by confocal microscopy in knee-surgery-obtained rheumatoid synovium.

**Conclusions:**

Combining our findings with previous data showing circulating pre-inflammatory mesenchymal cells preceding rheumatoid arthritis flares, we propose that a chemokine-orchestrated process, that seems to not being affected by short-term Disease-Modifying Antirheumatic Drug (DMARD) treatment, may contribute to ‘arthritis spreading’ in both RA and PsA. In this chronic process synovial fibroblasts and fibrocytes with pathogenic potential may migrate into distant synovium through CDH11-mediated homotypic binding.

## 1 Introduction

Rheumatoid arthritis (RA) and psoriatic arthritis (PsA) are chronic (auto)immune-mediated diseases characterized by peripheral joint inflammation, affecting more than 1% of the adult population (1, 2). Both share common pathogenetic mechanisms, but the role of adaptive immunity seems less important in patients with PsA and seronegative RA (3–6). Despite significant advances in the management of RA and PsA with biologic therapies against tumor necrosis factor (anti-TNF) (7–10), the pathogenetic mechanisms driving the initiation and perpetuation of joint inflammation remain unclear (4, 11, 12). Efforts to understand the dynamic cellular interactions involved in these mechanisms include searching for circulating synovial fibroblasts in the peripheral blood within the concept of cell migration. The pathogenic behavior of migrating synovial fibroblasts was first introduced in 2003 by Aidinis et al. (13), and was further supported by other studies (14, 15). More recent evidence in patients with RA suggested the presence of circulating pre-inflammatory mesenchymal (PRIME) cells prior to disease flare, possessing features of inflammatory synovial fibroblasts (16). Building upon these findings, a model wherein Pre-inflammatory Mesenchymal (PRIME) cells undergo activation by B cells in the weeks preceding a clinical flare in RA, and subsequently migrate from the periphery into the synovium, has been proposed (16).

Our previous investigations have shown elevated cadherin-11 (CDH11) mRNA transcripts, as well as rare non-hematopoietic cells expressing CDH11 in the blood of RA patients, which correlate with the concomitant presence of active polyarthritis (17). CDH11, a cell-cell adhesion protein, traditionally expressed by synovial fibroblasts, contributes to synovial hyperplasia and joint inflammation in arthritis (18, 19). CDH11 expression on rare circulating and tissue-resident leukocytes (17, 20–23) suggest a dynamic mesenchymal and hematopoietic cell crosstalk in chronic inflammatory arthritis.

Fibrocytes, bone marrow-derived progenitor cells that co-express hematopoietic stem cell antigens and can produce extracellular matrix components (24, 25), may also contribute to RA progression (24, 26–28) through diverse mechanisms, including the secretion of proinflammatory cytokines, antigen presentation, endothelial interactions, as well as differentiation into fibroblast-like synoviocytes (29). Studies on circulating fibroblasts and fibrocytes have not been conducted in patients with PsA, in contrast to those with RA (16, 26, 27, 29, 30). On the other hand, within the inflamed joint, fibroblast-like synoviocytes have indeed been shown to interact with infiltrating leukocytes to regulate inflammation and tissue damage in both RA and PsA (31–33).

Mass cytometry (CyTOF) has emerged as an ideal technology for identifying and characterizing rare cells in the blood, specifically those at concentrations of 1–2 cells per ml, due to its ability to simultaneously analyze multiple parameters at single-cell level with minimal background noise and enhanced sensitivity (34–36). Thus, by exploiting the high-dimensional power of single-cell proteomics, we aimed to identify and characterize fibroblasts, fibrocytes and leukocytes expressing CDH11 and/or other mesenchymal markers in active RA and PsA patient peripheral blood, comparing with controls and paired three-month post-antirheumatic treatment samples. Co-localization of CDH11-infiltrating leukocytes and fibroblasts in RA’s synovial tissue was also investigated.

## 2 Materials and methods

### 2.1 Patient enrollment

A total of 23 consecutive patients with moderately or highly active chronic inflammatory arthritis who attended the outpatient rheumatology clinics (**Supplementary Table S1**) were recruited. There were 11 patients with active RA (RA 2010 criteria), of whom 9 were seropositive (positive for rheumatoid factor (RF) and/or anti-citrullinated protein antibodies (ACPA)) and 2 were seronegative (negative for RF and ACPA), and 12 patients with active PsA (CASPAR criteria). Moderate to high disease activity was defined as DAS28-ESR> 3.2 for RA (active RA) and as DAPSA> 14 and/or ASDAS> 1.2 (for those having axial disease confirmed by X-rays or magnetic resonance) for PsA (active PsA). Exclusion criteria included prior treatment with the B-cell-depleting rituximab or chemotherapy, those with a history of solid or hematological neoplasms, as well as individuals who had been vaccinated within the preceding two weeks and those having clinical evidence of concurrent infection. A total of 9 healthy age and sex-matched individuals served as controls.

All patients, but one with PsA, were re-evaluated three months after the initiation or modification of antirheumatic treatment regimens for active disease, following standard clinical practice in accordance with national and international guidelines. Specifically, 8 patients received conventional synthetic disease-modifying antirheumatic drugs (csDMARDs; RA, n=3; PsA, n=5), and 14 patients received biologic/targeted-synthetic DMARDs (b/tsDMARDs; RA, n=8; PsA, n=6).

Mass cytometry was performed on peripheral blood samples at baseline and at the three-month timepoint. In addition, matched bone marrow samples were collected and analyzed from two PsA patients, one with inactive PsA and hypergammaglobulinemia, and one with active PsA and monoclonal gammopathy of undetermined significance (MGUS).

Confocal microscopy was employed in archival synovial tissues derived from four additional patients with RA undergoing knee joint arthroplasty. These patients were not included in the cohort analyzed by mass cytometry. Three patients with osteoarthritis (OA) served as controls.

The study complied with the Ethical Principles for Medical Research Involving Human Subjects according to the World Medical Association Declaration of Helsinki and the Oviedo Convention. Approval was obtained from the local Ethics and Scientific Committees of the University Hospitals of the National and Kapodistrian University of Athens (No. 314/2021). Written informed consent was obtained from all participating individuals.

### 2.2 Mass cytometry

#### 2.2.1 Whole blood and bone marrow staining

Peripheral blood was obtained by venipuncture and stored in ethylenediaminetetraacetic acid (EDTA) tubes for subsequent analysis by high-dimensional mass cytometry. To prevent potential contamination from skin-derived fibroblasts in our analysis, the initial 5 ml of blood collected were discarded. No other intervention was performed, and clinical data were collected from the patients’ files, as recorded during their regular visits to the Rheumatology Unit. To minimize bias, all samples were assigned a unique anonymized sample ID at the time of collection. These IDs were used consistently throughout experimental procedures and subsequent data analysis. The investigator performing the experiments and analyses was blinded to group allocation, as the sample IDs did not reveal group identity. Group assignments were only disclosed at the final stage of analysis, solely for the purpose of statistical comparison.

One ml of whole blood was incubated with heparin (at a final concentration of 100 U/ml) for 20 min at room temperature (RT). Next, 270 μl of heparin-blocked blood were incubated into the tube containing the dry antibody pellet of the Standard BioTools Maxpar Direct Immune Profiling Assay (MDIPA, Catalogue number: 201334) supplemented by the addition of antibodies against CDH11, PDPN, CD90 (or Thy-1), CD34 and Notch3, henceforth referred to as mesenchymal markers (**Supplementary Table S2**) for 30 min at RT. Then, 250 μl of lysing solution (1:10 dilution of BD FACS Lysing Solution, BD Biosciences, San Jose, CA, USA, in Maxpar Water) were added for red blood cell lysis, followed by incubation for 10 min at RT in the dark. Following the addition and incubation of 3 ml of Maxpar Water for 10 min at RT in the dark, the tube was centrifuged at 300 x g for 5 min and the supernatant was carefully aspirated. Cells were washed 3 times using 3 ml of Maxpar Cell Staining Buffer (CSB) and centrifuged at 300 x g for 5 min.

The bone marrow samples were resuspended and incubated in 10x volume of lysing solution for 10 min at RT. Following centrifugation at 350 x g for 5 min at 4°C, the supernatant was carefully aspirated, and then lysis was repeated. The pellet was then resuspended in 1 ml of cold Maxpar Phosphate Buffer Solution (PBS) and filtered through a 70 μm cell strainer. Cells were washed using 4 ml cold PBS and centrifuged at 350 x g for 5 min at 4°C. The pellet was then resuspended in 1 ml of CSB and cells were counted. A maximum of 5 million cells were aliquoted and centrifuged at 350 x g for 5 min at 4°C. After carefully aspirating the supernatant, the pellet was resuspended in 50 μl CSB. FcR-blocking was achieved by adding 5 μl of Human TruStain FcX (Biolegend, San Diego, CA, USA). After incubating for 10 min at RT, 215 μl of CSB were added, for a final volume of 270 μl. Then, the 270 μl were transferred into the tube containing the dry antibody pellet of MDIPA, supplemented by the addition of antibodies against CDH11, PDPN, CD90, CD34, and Notch3 for 30 min at RT. Cells were then washed twice by adding 3 ml CSB and were centrifuged at 300 x g for 5 min.

Next, cells from both whole blood and bone marrow samples were fixed and permeabilized by adding 1 ml of 1.6% freshly prepared formaldehyde solution in Maxpar PBS. After incubating for 10 min at RT, the tube was centrifuged at 800 x g for 5 min and the supernatant was carefully aspirated. Finally, DNA staining was performed by incubating the cell pellet with 1 ml of 125 nM Cell-ID Intercalator-Ir solution in Maxpar Fix and Perm Buffer and incubating at 4°C overnight. All antibodies and buffers were purchased from Standard BioTools Inc., San Francisco, CA, USA, unless otherwise stated.

#### 2.2.2 Mass cytometry data acquisition and processing

Sample acquisition was conducted using a 3^rd^ generation Helios mass cytometer. Prior to acquisition, the instrument was first tuned with Tuning Solution and a bead sensitivity test was performed using EQ Four Element Calibration Beads. Samples were washed twice with 1 ml of Maxpar CSB and twice with 1 ml of Cell Acquisition Solution (CAS). Subsequently, cells were resuspended in CAS containing 0.1X EQ Four Element Calibration Beads, to a maximum cell concentration of 1 x 10^6^ cells/ml. Finally, cells were filtered through a 35-mm nylon mesh cell strainer (BD Biosciences, San Jose, CA, USA). Using the CyTOF Software version 7.0.8493, an average of 500,000 events were acquired per file, at a flow rate of 300–500 cells/sec. The generated FCS files were normalized using the CyTOF software and then Cytobank (Beckman Coulter Life Sciences, Indianapolis, IN, USA) was used for further processing and analysis.

The gating strategy employed for the initial pre-processing stages is shown in **Supplementary Figure S1**. The phenotype of circulating fibrocytes and fibroblasts was based on previous studies (16, 22, 27, 37). Specifically, fibroblasts were defined as PDPN^+^CD45^-^ cells strictly gated in lineage-negative lymphoid and granulocytic cells (CD3^-^CD19^-^CD4^-^CD8^-^CD56^-^CD66b^-^CD294^-^). Based on all the possible combinations of the mesenchymal markers CDH11, CD34, CD90 and Notch3, the presence of a total of 16 fibroblast phenotypes was investigated (**Supplementary Table S3**). Similarly, fibrocytes were defined as CD34^+^HLA-DR^+^CD45^+^ cells strictly gated in lineage-negative lymphoid and granulocytic cells (CD3^-^CD19^-^CD4^-^CD8^-^CD56^-^CD66b^-^CD294^-^). Again, based on all the possible combinations of CDH11, PDPN, CD90 and Notch3, the presence of a total of 16 fibrocyte phenotypes was investigated (**Supplementary Table S4**). The presence of the chemokine receptors C-C chemokine receptor (CCR) 6, CCR7, C-X-C chemokine receptor (CXCR) 3 and CXCR5 was investigated in both fibroblasts and fibrocytes. In addition, 12 leukocyte subpopulations were gated and investigated for the co-expression of CD90 and CDH11. The phenotypes of the identified leukocyte subpopulations are listed in **Supplementary Table S5**. The detailed gating strategy for all cell populations is shown in **Supplementary Figures S2**-**18**.

To visualize the high-dimensional data in two dimensions, the dimensionality reduction algorithm tSNE-CUDA (GPU-accelerated t-distributed stochastic neighbor embedding) was used through Cytobank, based on the expression of the 30 surface markers of the MDIPA panel. For this analysis, 15,000 randomly selected cells from each sample were analyzed, totaling 810,000 cells.

### 2.3 Plasma proteomics using Olink technology

#### 2.3.1 Sample preparation and multiplex protein measurement

Matched plasma samples were collected from a subset of controls (n=7) and patients (RA, n=10; PsA, n=10) and analyzed using the Olink platform. After collecting the appropriate volume for mass cytometry staining, the remaining peripheral blood was centrifuged at 1000 × g for 10 min to separate plasma. The plasma samples were then further clarified by sequential centrifugation at 2000 × g for 10 min to remove cellular debris, followed by 10,000 × g for 30 min, and finally filtered through a 0.22 µm pre-wetted filter to eliminate residual platelets. The clarified plasma was then stored at −80°C until further use.

Plasma proteomic profiling was conducted using the Olink Explore 384 Inflammation panel (Olink Proteomics, Uppsala, Sweden), following the manufacturer’s instructions. This platform employs the Proximity Extension Assay (PEA) technology combined with Next-Generation Sequencing (NGS) for high-throughput protein quantification, enabling the simultaneous measurement of 384 proteins involved in inflammatory processes. Protein expression levels are reported in Normalized Protein eXpression (NPX) units, which are relative quantification values on a log2 scale. All data were normalized and intensity-calibrated using Olink’s standard procedures (NPX™ Map version 1.0.2) to adjust for intra-plate and technical variation. Only proteins that passed Olink’s internal quality control criteria, including detection above the assay limit of detection and absence of assay warnings, were included in the final analyses. Stringent quality control steps were applied throughout the workflow to ensure data reliability and minimize technical variance.

Proteomic data derived from the Olink platform were analyzed to identify differences between study groups and to assess associations between cell populations and protein expression levels. All analyses were conducted in the *R* programming environment (R version 4.4.2), while data visualization was performed using the *ggplot2* package (version 3.5.1). Biological interpretation of proteomics profiles into functional annotations, including Hallmarks (38, 39), KEGG pathways (40), and Gene Ontology (GO) terms (41), was performed through the Gene Set Enrichment Analysis (GSEA) using the *fgsea* package (version 1.32.0) (42).

### 2.4 Immunofluorescence staining and imaging analysis of synovial tissue

Synovial tissue was processed and cryopreserved as follows: each synovial tissue specimen was dissected into 1 to 5 mm fragments, then submerged in Cryostor CS10 (Sigma-Aldrich, St. Louis, MO, USA), and then stored at −80 °C prior to transfer to long-term storage in liquid nitrogen. Imaging of the RA tissue was carried out in the BSRC Al. Fleming Imaging facility on FFPE synovial tissue sections.

RA synovial tissues were first fixed in 4% PFA for at least 12 h followed by xylene and ethanol processing and embedded in paraffin. 5μm sections were cut onto histology slides using the manual microtome LeicaRM2125RTS. Prior to staining, sections were rehydrated, and antigen retrieved in 10 mM Sodium Citrate buffer (pH 6.0) for 20 min at 96 °C and further 20 min at RT. Slides were rehydrated with PBS before blocking with 10% normal donkey serum. Primary antibodies were then applied in PBS-10% serum, THY1/CD90 (sheep polyclonal, AF2067, Bio-Techne), CD45 (clone D3F8Q, Cell Signaling), PDPN (NZ-1.3, eBioscience, PE), CDH11 (MA1-06306, Thermo Fisher Scientific) and Ly6G (ab122501, Abcam). Appropriate isotype controls were used on separate sections. Secondary antibodies included Donkey anti-Rabbit IgG (H+L) Highly Cross-Adsorbed Secondary Antibody, Alexa Fluor™ 488 (Invitrogen- A-21206), Donkey anti-Sheep IgG (H+L) Cross-Adsorbed Secondary Antibody, Alexa Fluor™ 647 (Invitrogen, A-21448), Donkey anti-Mouse IgG (H+L) Highly Cross-Adsorbed Secondary Antibody, Alexa Fluor™ 647 (Invitrogen, A-31571), Goat anti-Mouse IgG (H+L) Highly Cross-Adsorbed Secondary Antibody, Alexa Fluor™ 594 (Invitrogen, A-11032). Primary antibodies were titrated and used at a concentration that ranged from 1:100 to 1:1000, depending on the antibody, whereas secondary antibodies were all used at 1:400 dilution. Slides were mounted in prolong diamond (Thermo Fisher Scientific, Waltham, MA, USA) and stored at 4 °C before imaging.

Images were obtained using a Leica TCS SP8X White Light Laser confocal system in linear deconvolution or sequential mode and were analyzed using ImageJ (FIJI, publicly available NCBI software).

### 2.5 Statistical analysis

Given the exploratory nature of the study, proposing a statistical hypothesis to determine the appropriate sample size was not possible. Data are presented as median, lower and upper quartiles unless otherwise stated. Statistical analysis was performed using GraphPad Prism version 9.0.0 (GraphPad Software, San Diego, CA, USA). Normal distribution was assessed using the D’Agostino-Pearson normality test. For normally distributed data, homogeneity of variances was tested using either the F-test or Bartlett’s test. Fisher’s exact test was employed for comparing categorical variables. Comparisons of numerical values between the two groups were performed using an unpaired t-test or Mann-Whitney U test, as appropriate. Comparisons of numerical values between multiple groups were conducted using One-Way ANOVA or a Kruskal–Wallis test, followed by the two-stage step-up method of Benjamini, Krieger and Yekutieli for *post-hoc* analysis. Paired comparisons were performed using the Wilcoxon test. Correlation coefficients between variables were calculated according to Spearman’s rank correlation coefficient and were performed using IBM SPSS Statistics 28.0.0.0 (IBM, Armonk, NY, USA). The level of significance was established at a p-value< 0.05 for two-sided tests.

Regarding the statistical analysis of Olink results, differential protein expression was assessed using the *limma* package (version 3.62.1) (43). Proteins with an adjusted p-value below 0.05 following Benjamini-Hochberg correction and an absolute log2 fold change (log2FC) greater than 1 were considered Differentially Expressed Proteins (DEPs). Pathway enrichment analysis was performed using the t-test statistic from the differential protein expression analysis, with pathways being considered significantly enriched for an adjusted p-value below 0.05, after Benjamini-Hochberg correction. Additionally, Pearson correlation analysis was conducted to evaluate associations between protein NPX levels and cell population counts. Only proteins involved in statistically significant pathways from the GSEA were included in this analysis. Correlation p-values have been corrected for multiple comparisons using the Benjamini-Hochberg method, with a significance threshold set at an adjusted p-value of 0.1.

## 3 Results

### 3.1 Circulating fibroblasts expressing CDH11, CD90, HLA-DR and CCR7 characterize RA and PsA

**Figure 1:**
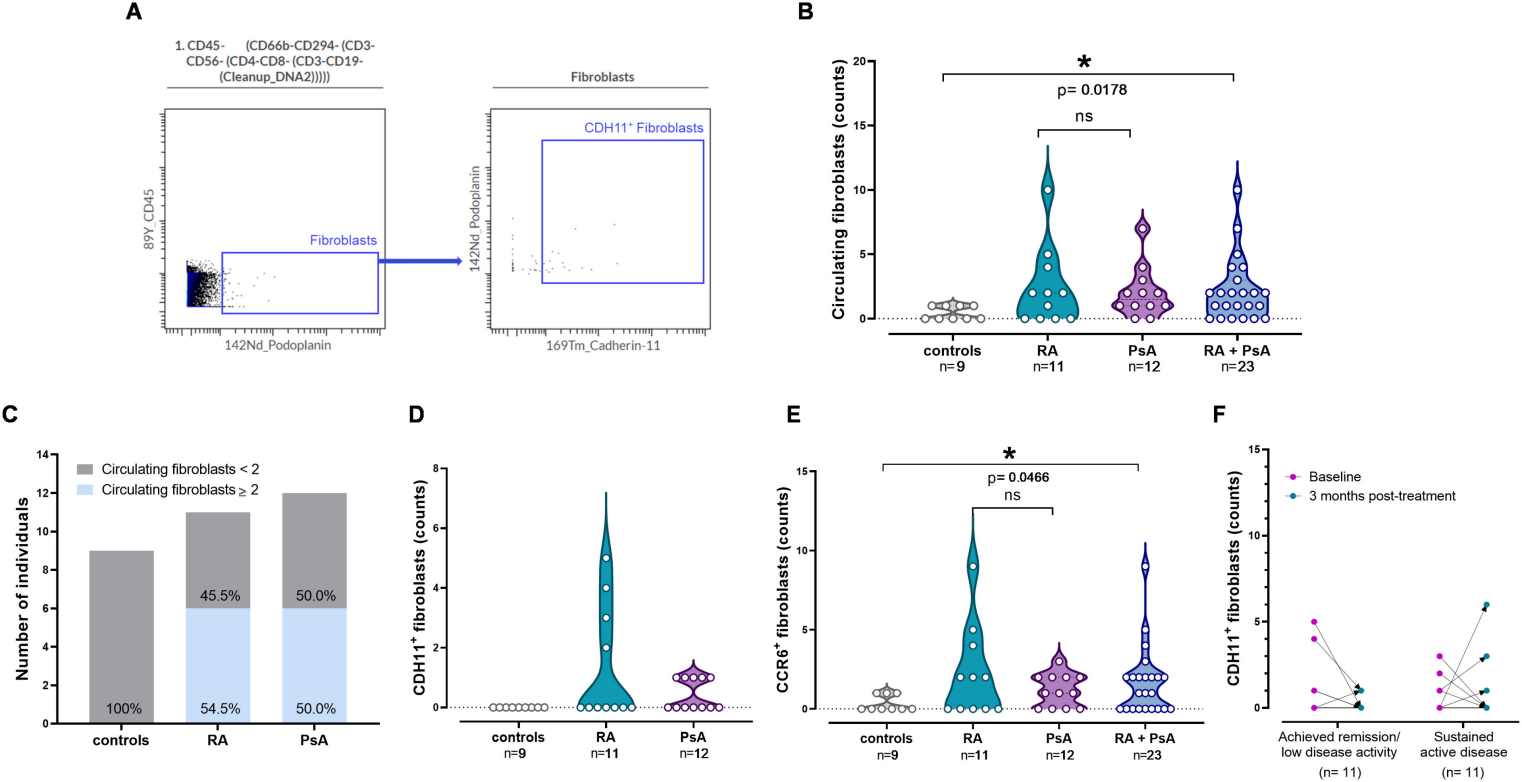
Elevated counts of circulating fibroblasts in patients. **(A)** Manual gating of CDH11^+^ fibroblasts, depicted in concatenated dot plots (n=32). **(B)** Violin plots showing the counts of circulating fibroblasts in patient groups and controls. Each point corresponds to an individual. Horizontal dotted lines represent the median (middle line) and the 25^th^ (lower line) and 75^th^ (upper line) percentiles. Groups were compared using Kruskal–Wallis test, followed by the two-stage step-up method of Benjamini, Krieger and Yekutieli for *post-hoc* analysis (HC vs RA vs PsA) and Mann-Whitney U test (HC vs merged RA+PsA). Asterisks denote statistically significant differences between groups. **(C)** Bar plots showing the number of individuals in the samples for whom the counts of detected circulating fibroblasts were equal to or greater than two (grey bar = fibroblast counts < 2 per sample; blue bar = fibroblast counts ≥ 2 per sample). **(D)** Violin plots showing the counts of CDH11^+^ circulating fibroblasts in patient groups and controls. Each point corresponds to an individual. Horizontal dotted lines represent the median (middle line) and the 25^th^ (lower line) and 75^th^ (upper line) percentiles. Groups were compared using Kruskal–Wallis test, followed by the two-stage step-up method of Benjamini, Krieger and Yekutieli for *post-hoc* analysis. **(E)** Violin plots showing the counts of CCR6^+^ circulating fibroblasts in patient groups and controls. Each point corresponds to an individual. Horizontal dotted lines represent the median (middle line) and the 25^th^ (lower line) and 75^th^ (upper line) percentiles. Groups were compared using Kruskal–Wallis test, followed by the two-stage step-up method of Benjamini, Krieger and Yekutieli for *post-hoc* analysis (HC vs RA vs PsA) and Mann-Whitney U test (HC vs merged RA+PsA). Asterisks denote statistically significant differences between groups. (grey plot= controls, n= 9; cyan plot= RA, n= 11; purple plot= PsA, n= 12; blue plot= RA + PsA, n= 23). **(F)** CDH11^+^ fibroblast counts in patients who achieved remission (n=11) or remained clinically active (n=11) post-treatment, measured at baseline (magenta points) and in their three-month follow-up (cyan points). Each point corresponds to an individual patient. Comparison of paired samples between time-points was performed using the Wilcoxon test. (n, number of individuals; RA, rheumatoid arthritis; PsA, psoriatic arthritis; CDH11, cadherin-11; ns, not significant; *p< 0.050).

Circulating fibroblasts, i.e., non-hematopoietic cells expressing podoplanin (PDPN^+^CD45^-^CD3^-^CD19^-^CD4^-^CD8^-^CD56^-^CD66b^-^CD294^-^) (16, 22) (**Figure 1A**), were identified in both active RA and PsA patients by mass cytometry. Specifically, a total of 26 circulating fibroblasts were detected in 7/11 RA patients (67%), representing both seropositive and seronegative RA patients and a total of 24 circulating fibroblasts were detected in 10/12 PsA patients (83%), with an estimated median frequency of 7 cells/ml (**Supplementary Tables S6**, **S7**). In contrast to patients with RA and PsA, a total of only 4 circulating fibroblasts were detected in 4/9 control individuals (44%) (p=0.018, **Figure 1B**). No specific clinical characteristics were associated with increased levels of detectable circulating fibroblasts in patients. Moreover, two or more circulating fibroblasts/sample were observed in 55% and 50% of active RA and PsA patients, respectively, contrasting with only one circulating fibroblast per control sample (p= 0.012; **Figure 1C**).

We also investigated the expression of CDH11, CD34, CD90 and Notch3 on the identified PDPN-expressing circulating fibroblasts at baseline. These four proteins characterize mesenchymal cells in general, and their expression has been associated with higher cell motility, adhesion, migration and invasion (18, 19, 44–46). Notably, CDH11 and CD90 were expressed only by patient-derived circulating fibroblasts, with higher frequency in RA than PsA patients (**Figure 1D**; **Supplementary Figure S19A**). Moreover, expression of HLA-DR, denoting antigen-presenting properties, was exclusively noted on patients’ circulating fibroblasts (**Supplementary Figure S19A**). By exploring all potential combinations involving CDH11, CD34, CD90, and Notch3, a total of 16 distinct fibroblastic subsets were investigated (FB1-16; **Supplementary Table S3**). Out of these 16 phenotypes, 7 were identified in RA (FB1,2,3,7,8,12,16) and 6 in PsA (FB1,2,5,7,12,16) versus only 2 in controls (FB1,9; **Supplementary Figure 2A**).

Next, we examined the presence of a panel of chemokine receptors (CCR4, CCR6, CCR7, CXCR3 and CXCR5), known to orchestrate migration of cells in peripheral tissues, on the identified circulating fibroblasts. Notably, CCR7 was observed only in patient-derived circulating fibroblasts, suggesting their potential trafficking toward lymphoid organs or the inflamed joint (**Supplementary Figure S19B**). Moreover, circulating fibroblasts primarily expressed CCR6 (**Figure 1E**), with CCR6^+^ fibroblast counts being elevated in patients compared to controls (p=0.047; **Supplementary Figure S19B**).

**Figure 2:**
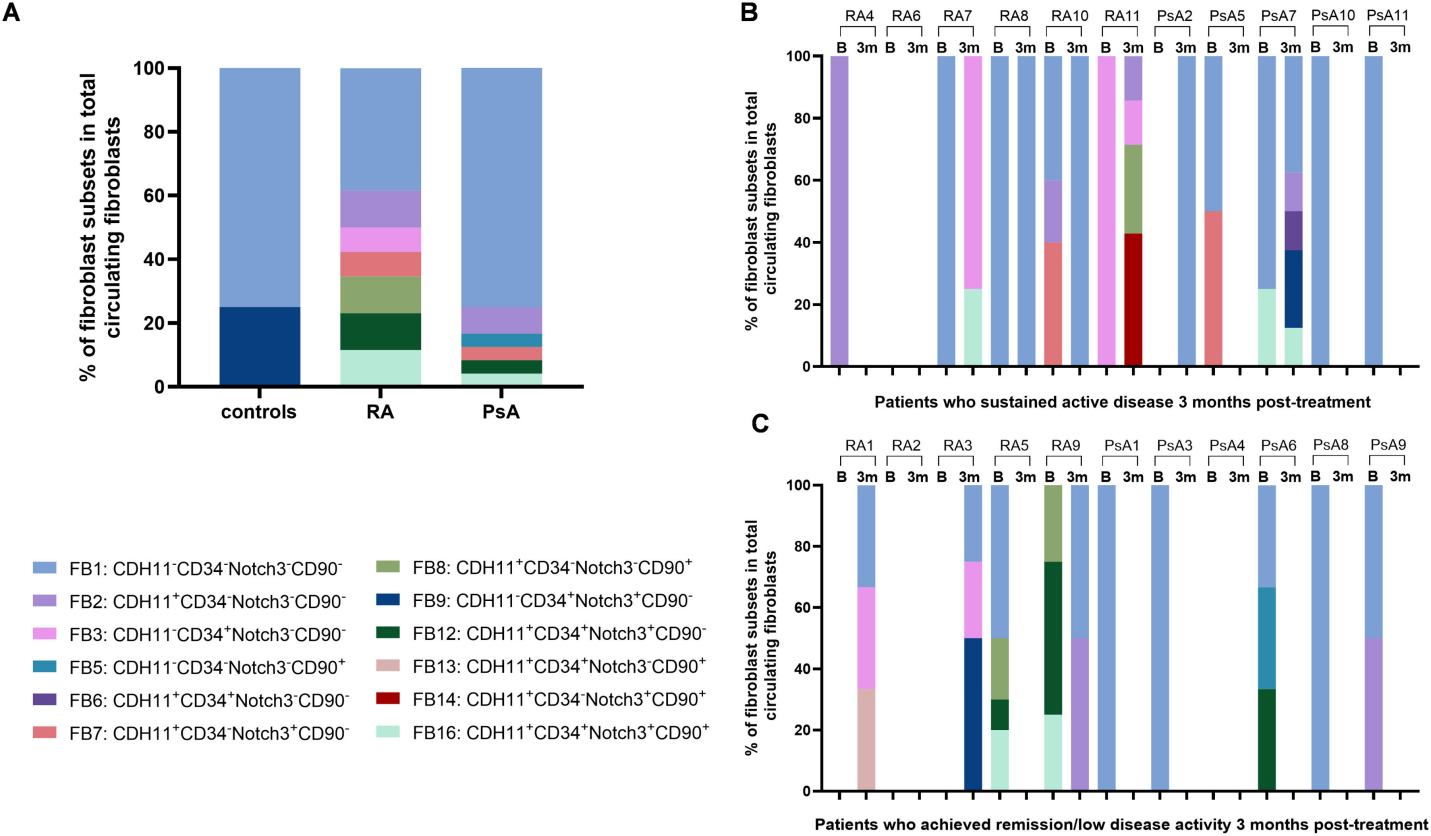
Phenotypic heterogeneity of circulating fibroblasts in terms of mesenchymal marker combinations. **(A)** Bar plots showing the proportion of identified mesenchymal marker combination phenotypes within total detected circulating fibroblasts per study group, at baseline. Each color represents a different phenotype, as indicated on the right. **(B)** Bar plots depicting the proportion of identified mesenchymal marker combination phenotypes within the fibroblasts detected in each sample, at baseline and three months post-treatment, in patients (RA, n=4; PsA, n=5) who sustained active arthritis in their three-month follow-up. **(C)** Bar plots showing the proportion of identified mesenchymal marker combination phenotypes within the fibroblasts detected in each sample, at baseline and three months post-treatment, in patients (RA, n=5; PsA, n=4) who achieved clinical remission in their three-month follow-up. Each color represents a different subset, as indicated on the top right. (n, number of individuals; RA, rheumatoid arthritis; PsA, psoriatic arthritis; CDH11, cadherin-11; B = baseline; 3m = 3 months post-treatment).

Following three months of antirheumatic treatment, a total of 38 circulating fibroblasts were detected in 10/22 patients (46%), at comparable frequencies and having similar characteristics to those identified at baseline. Among the re-evaluated patients, there were 11/22 who achieved clinical remission/low disease activity (5 RA and 6 PsA patients), while the remaining 11 patients had persistent active disease, albeit less active than baseline. In these repeated measurements, 11 and 27 circulating fibroblasts were detected in the peripheral blood of 4/11 patients in remission and 6/11 patients with persistently active disease, respectively (**Supplementary Table S6**; **Supplementary Figure S20**). The levels of CDH11^+^ fibroblasts remained elevated in the peripheral blood of both RA and PsA patients, regardless of clinical responses (**Figure 1F**). Fibroblast phenotypic composition in terms of mesenchymal marker expression demonstrated inter-patient and intra-patient variability between the two time-points (**Figures 2B, C**). Of note, circulating fibroblasts co-expressing all examined mesenchymal markers (FB16) were detected only in active patients (**Figures 2B, C**), indicating potential relevance to disease activity.

Finally, to gain insight into the potential origin of circulating fibroblasts, we further analyzed paired samples of peripheral blood and bone marrow from 2 PsA patients. Rare fibroblasts characterized by CD34 expression were indeed present in the bone marrow, however, none expressed CDH11. These findings suggest that circulating fibroblasts expressing CDH11 and exclusively found in patients with chronic arthritis are more likely to be derived from inflamed joints than from the bone marrow.

### 3.2 Circulating CDH11^+^ and CCR7^+^ fibrocytes are increased in RA and PsA

**Figure 3:**
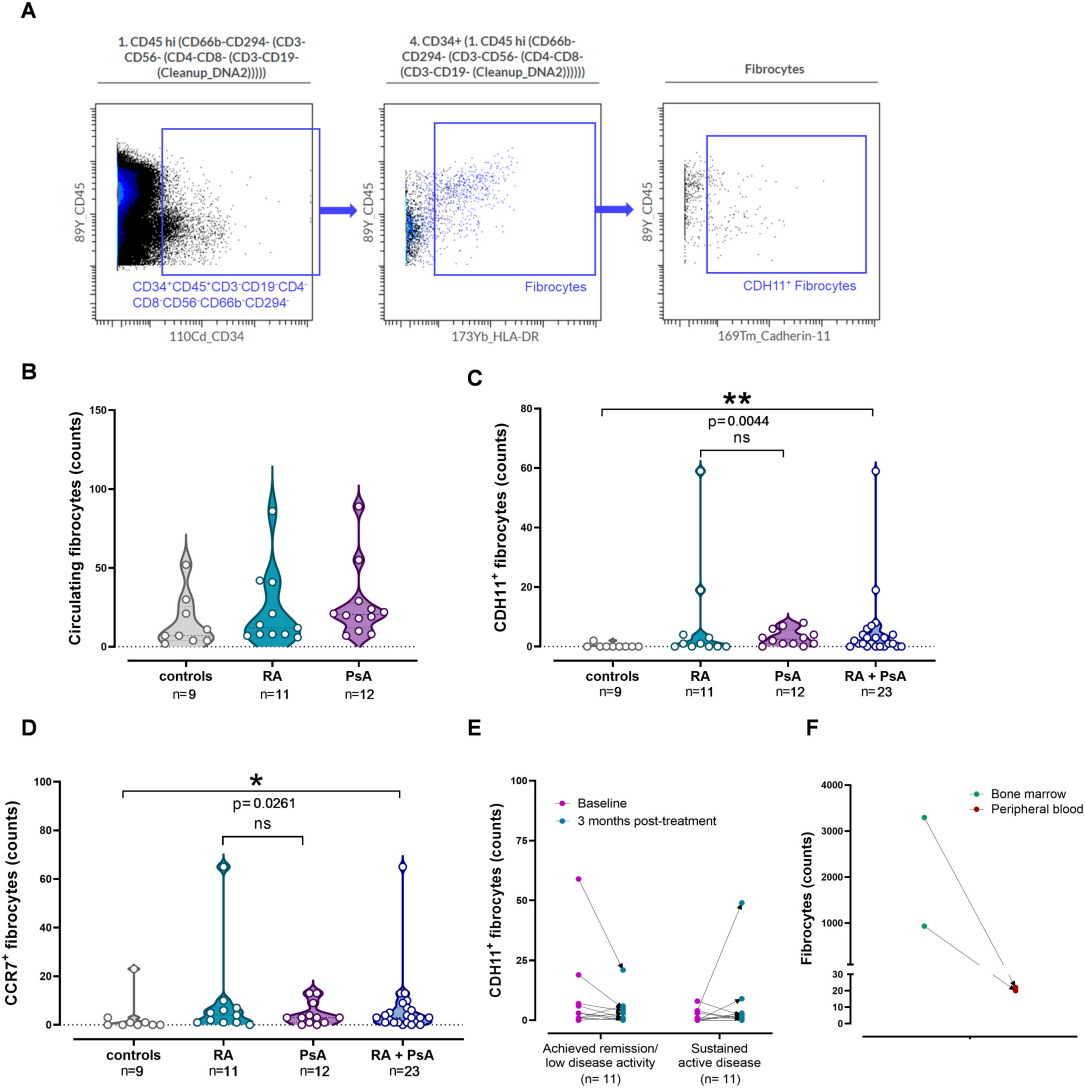
Elevated counts of circulating fibrocytes with migratory potential in patients. **(A)** Manual gating of CDH11^+^ fibrocytes, depicted in concatenated dot plots (n=32). **(B)** Violin plots showing the counts of circulating fibrocytes across the study groups. Each point corresponds to an individual. **(C)** Violin plots showing the counts of CDH11^+^ fibrocytes in patient groups and controls. Each point corresponds to an individual. **(D)** Violin plots show the counts of CCR7^+^ fibrocytes in patient groups and controls, while no differences were observed between RA and PsA. Each point corresponds to an individual (grey plot= controls, n= 9; cyan plot= RA, n= 11; purple plot= PsA, n= 12; blue plot= RA + PsA, n= 23). Horizontal dotted lines represent the median (middle line) and the 25^th^ (lower line) and 75^th^ (upper line) percentiles. For B, C, and D, groups were compared using Kruskal–Wallis test, followed by the two-stage step-up method of Benjamini, Krieger and Yekutieli for *post-hoc* analysis (HC vs RA vs PsA) and Mann-Whitney U test (HC vs merged RA+PsA). Asterisks denote statistically significant differences between groups. **(E)** CDH11^+^ fibrocyte counts in patients who achieved remission (n=11) or remained clinically active (n=11) post-treatment, measured at baseline (magenta points) and in their three-month follow-up (cyan points). Comparison of paired samples between time-points was performed using the Wilcoxon test. **(F)** Fibrocyte counts in the peripheral blood (green points) and matched bone marrow aspirates (red points) from 2 PsA patients. (n, number of individuals; RA, rheumatoid arthritis; PsA, psoriatic arthritis; CDH11, cadherin-11; ns, not significant; *p< 0.050; **p≤ 0.010).

**Figure 4:**
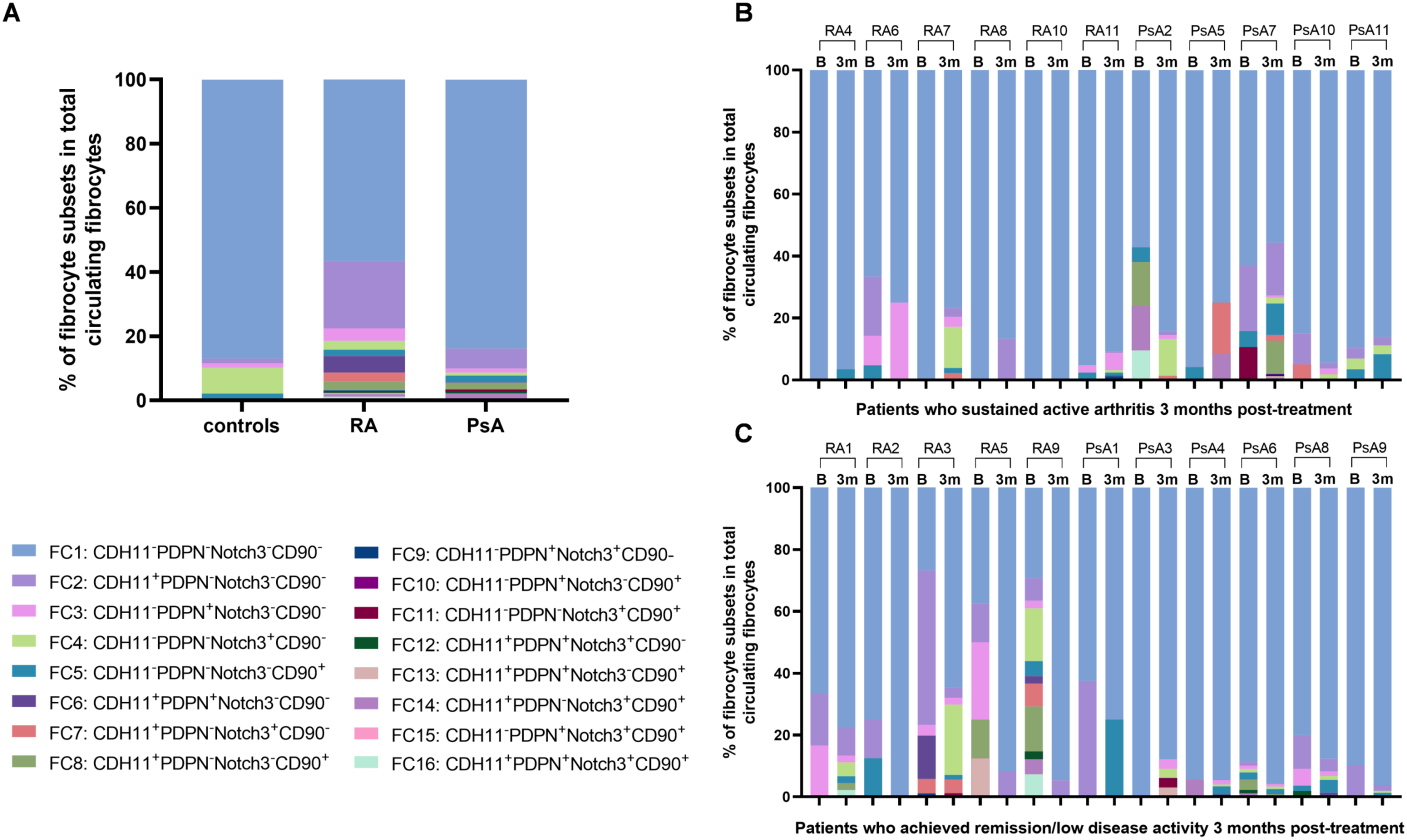
Phenotypic heterogeneity of circulating fibrocytes in terms of mesenchymal marker combinations. **(A)** Bar plots showing the proportion of identified mesenchymal marker combination subsets within total detected circulating fibrocytes per study group, at baseline. **(B)** Bar plots depicting the proportion of identified mesenchymal marker combination subsets within the fibrocytes detected in each sample, at baseline and three months post-treatment, in patients (RA, n=4; PsA, n=5) who sustained active arthritis in their three-month follow-up. **(C)** Bar plots showing the proportion of identified mesenchymal marker combination subsets within the fibrocytes detected in each sample, at baseline and three months post-treatment, in patients (RA, n=5; PsA, n=4) who achieved clinical remission in their three-month follow-up. Each color represents a different phenotype, as indicated on the top right. (n, number of individuals; RA, rheumatoid arthritis; PsA, psoriatic arthritis; CDH11, cadherin-11; PDPN, podoplanin; B = baseline; 3m = 3 months post-treatment).

Circulating fibrocytes (CD34^+^HLA-DR^+^CD45^+^CD3^-^CD19^-^CD4^-^CD8^-^CD56^-^CD66b^-^CD294^-^) (27, 37) (**Figure 3A**) were detected in all examined blood samples from patients and controls. A total of 253 circulating fibrocytes were detected in RA (range, 6-86) and 322 in PsA patients (range, 7-89), with an estimated median frequency of 70 cells/ml, versus a total of 138 (range, 2-52) circulating fibrocytes in controls (**Figure 3B**, **Supplementary Table S6**). Certain circulating fibrocytes expressed CDH11, PDPN, Notch3, or CD90, with higher percentages detected in RA, followed by PsA patients, compared to controls (**Supplementary Figure S21A**). Of them, a significant elevation of CDH11^+^ fibrocyte counts was found in patients compared to controls (p= 0.004), as shown in **Figure 3C**. Through the examination of all potential combinations involving CDH11, PDPN, CD90, and Notch3 in our fibrocyte pool, a total of 16 subsets were formed (FC1-16; **Supplementary Table S4**). Out of these distinct phenotypes, 13 and 11 were identified in RA and PsA patients, respectively, versus only 5 (FC1-5) in controls (**Figure 4A**). No co-expression of the examined mesenchymal markers (FC6-16) was detected in controls’ fibrocytes.

Regarding chemokine receptor expression on circulating fibrocytes, we found that these cells predominantly expressed the chemokine receptor CCR6, possibly facilitating their recruitment into sites of inflammation (**Supplementary Figure S21B**), and to a lesser extent CCR7 and CCR4. Interestingly, patients exhibited increased counts of circulating CCR7^+^ fibrocytes compared to controls (p=0.026; **Figure 3D**).

Upon patients’ re-evaluation after three months of antirheumatic treatment, circulating fibrocytes were increased compared to baseline, both in patients who achieved remission (p=0.008) and in patients with persistent active disease (p=0.019), regardless of RA or PsA (**Supplementary Figure S22**). As in the case of CDH11^+^ fibroblasts, CDH11^+^ fibrocyte counts remained elevated three months post-treatment, regardless of the clinical status of the patients (**Figure 3E**). Moreover, the phenotypic composition of fibrocytes in terms of mesenchymal marker combinations displayed interpatient and intrapatient variability between the two time-points (**Figures 4B, C**).

Finally, highly elevated fibrocyte counts were evident in bone marrow aspirates when compared to the matched blood samples in two patients with PsA. Indeed, fibrocyte counts were increased by 50 to 100 times compared to the peripheral blood (**Figure 3F**), indicating that circulating fibrocytes originate primarily from bone marrow.

### 3.3 Neutrophils co-expressing CDH11, CD90 and CCR6 are increased in RA and PsA

**Figure 5:**
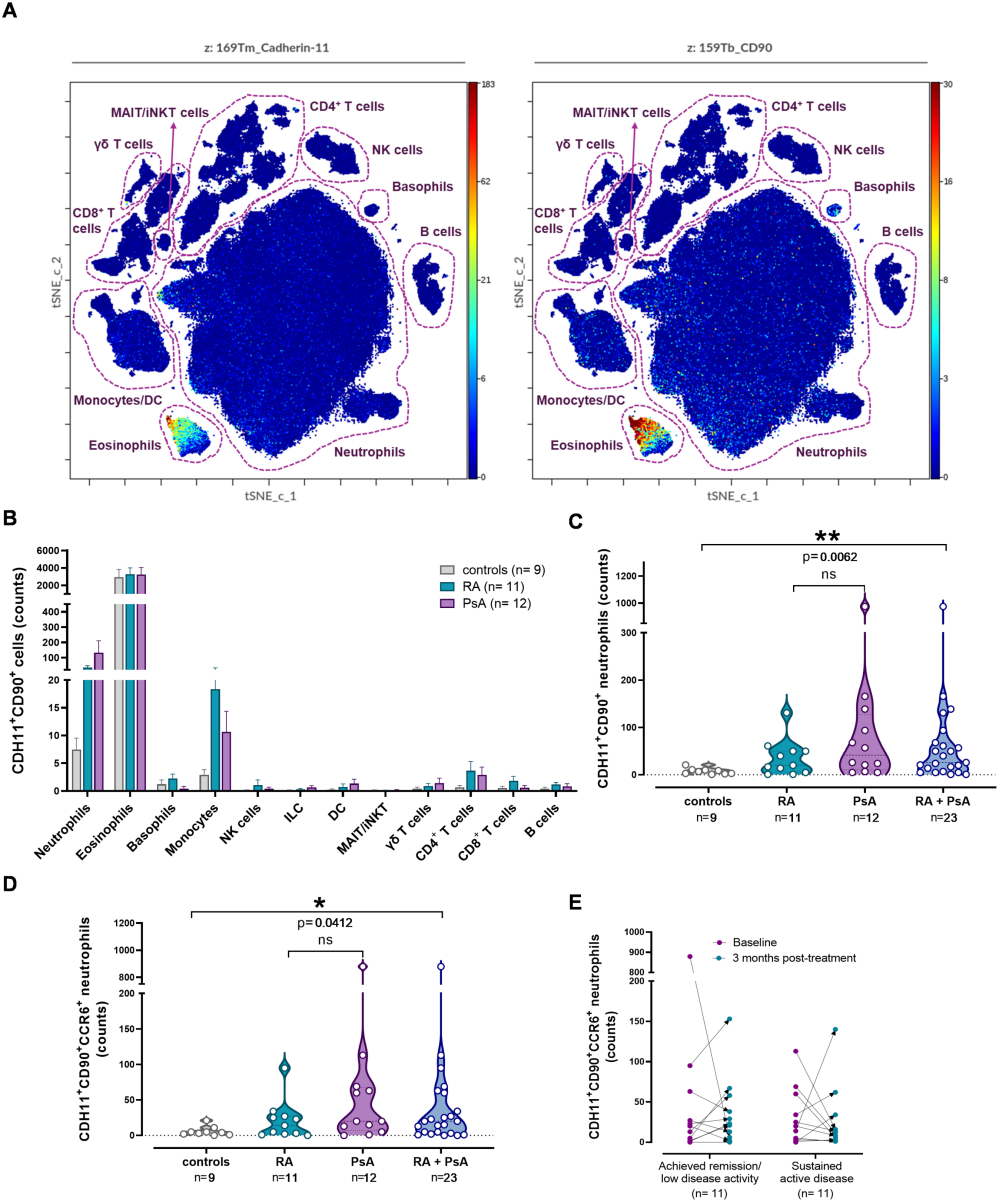
Increased counts of neutrophils co-expressing CDH11 and CD90 in patients as compared to controls. **(A)** tSNE-CUDA plot shows 810,000 randomly selected CD45^+^ cells in the 2-dimensional space. Leukocyte subpopulations are circled with a dotted line on the tSNE-CUDA plot. Each dot represents a cell and is colored according to CDH11 and CD90 intensity on a spectrum heat scale (red = high intensity; blue = low intensity). Arcsine-transformed color scales report the raw values of the marker’s intensity. **(B)** Bar plots depicting the counts of CDH11^+^CD90^+^ cells per leukocyte subpopulation across the study groups (mean ± SEM). **(C)** Violin plots showing the counts of CDH11^+^CD90^+^ neutrophils in patient groups and controls. Each point corresponds to an individual. **(D)** Violin plots showing the counts of CDH11^+^CD90^+^ neutrophils expressing CCR6, in patient groups and controls. Each point corresponds to an individual (grey plot= controls, n= 9; cyan plot= RA, n= 11; purple plot= PsA, n= 12; blue plot= RA + PsA, n= 23). Horizontal dotted lines represent the median (middle line) and the 25^th^ (lower line) and 75^th^ (upper line) percentiles. For C, and D, groups were compared using Kruskal–Wallis test, followed by the two-stage step-up method of Benjamini, Krieger and Yekutieli for *post-hoc* analysis (HC vs RA vs PsA) and Mann-Whitney U test (HC vs merged RA+PsA). Asterisks denote statistically significant differences between groups. **(E)** CDH11^+^CD90^+^ neutrophil counts in patients in remission (n=11) and those with active arthritis (n=11) post-treatment, measured at baseline and in their three-month follow-up. Each point corresponds to an individual patient (red points= baseline levels; cyan points= three months post-treatment levels). Comparison of paired samples between time-points was performed using the Wilcoxon test. (n, number of individuals; RA, rheumatoid arthritis; PsA, psoriatic arthritis; CDH11, cadherin-11; ILC, innate lymphoid cells; DC, dendritic cells; MAIT, musical-associated invariant T cells; iNKT, invariant natural killer T cells; ns, not significant; *p< 0.050; **p≤ 0.010).

To expand on our previous observations on CDH11 expression on circulating hematopoietic CD45^+^ cells (17), and given that CD90 may promote leukocyte extravasation (46, 47), we sought to investigate the expression of these mesenchymal cell markers on circulating leukocytes. Hematopoietic cells co-expressing CD90 and CDH11, which mediates cell-to-cell binding, were indeed identified by mass cytometry (**Figure 5A**; **Supplementary Figure S23**), comprising approximately 1% of total CD45^+^ cells in the peripheral blood, with comparable proportions across RA, PsA and controls. Further analysis revealed that co-expression of CDH11 and CD90 was present in all 12 tested major leukocyte subpopulations (neutrophils, eosinophils, basophils, monocytes, natural killer cells, dendritic cells, innate lymphoid cells, mucosal-associated invariant T cells/invariant natural killer cells, γδ T cells, CD4^+^ T cells, CD8^+^ T cells and B cells), with the highest counts being detected in cells of innate immunity, particularly eosinophils, followed by neutrophils and monocytes (**Figure 5B**). No significant differences were noted between patients and controls in all CDH11^+^CD90^+^leukocyte subpopulations except one. Specifically, CDH11^+^CD90^+^ neutrophils were found to be highly enriched in patients compared to controls (p=0.006; **Figure 5C**).

The expression of chemokine receptors on the surface of CDH11^+^CD90^+^ neutrophils was subsequently investigated (**Supplementary Figure S24**). Among the examined receptors, CDH11^+^CD90^+^CCR6^+^ neutrophil counts were significantly higher in patients compared to controls (p=0.041; **Figure 5D**; **Supplementary Figure S25**). All examined chemokine receptors were preferentially overexpressed on CD90^+^CDH11^+^ neutrophils as compared to CDH11^-^CD90^-^, CDH11^-^CD90^+^ and CDH11^+^CD90^-^ neutrophils (**Supplementary Figure S26**). As Notch3 is a receptor whose expression on immune cells has been associated with enhanced extravasation (48), we also examined its presence on CDH11^+^CD90^+^ neutrophils. Although our analysis revealed no significant differences in CDH11^+^CD90^+^Notch3^+^ neutrophil counts between patients and controls, investigation of chemokine receptor expression showed that CDH11^+^CD90^+^Notch3^+^ neutrophils co-expressing CCR6^+^ or CCR7^+^ were enriched in patients versus controls (p=0.027, and p=0.019, respectively; **Supplementary Figure S27**).

In repeated measurements upon patient re-evaluation following antirheumatic treatment, no significant differences were observed concerning CDH11^+^CD90^+^ leukocytes, despite changes in disease activity. Specifically, total CDH11^+^CD90^+^ neutrophil counts and CDH11^+^CD90^+^CCR6^+^ neutrophil counts remained elevated in both patients with persistent active disease and patients who achieved clinical remission/low disease activity (**Figure 5E**; **Supplementary Figure S28**).

Finally, we searched for individual correlations between CDH11^+^CD90^+^ leukocyte subpopulations and circulating fibroblasts and fibrocytes expressing CDH11 in patients with RA and PsA at baseline. No correlation was observed between circulating CDH11^+^ fibroblasts and CDH11^+^CD90^+^ leukocytes. Notably however, we found that the individual numbers of circulating fibrocytes expressing CDH11 correlated positively with the corresponding numbers of CDH11^+^CD90^+^ neutrophils (r_s_=0.498; p=0.015), as well as with monocytes, natural killer cells, innate lymphoid cells, dendritic cells and γδ T cells co-expressing CD90 and CDH11 (**Supplementary Table S8**); no correlations were found with CD4^+^ T, CD8^+^ T or B cells.

### 3.4 The presence of circulating CDH11^+^ fibroblasts is associated with distinct plasma proteomic signatures

**Figure 6:**
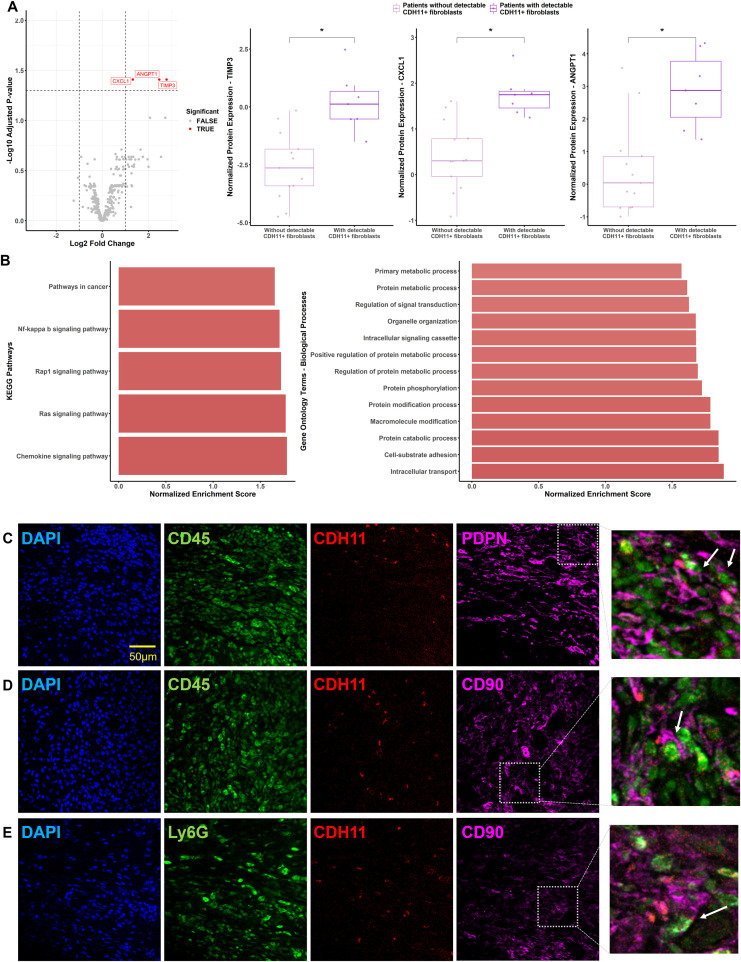
Circulating CDH11^+^ fibroblasts are associated with specific plasma protein signatures and enriched signaling pathways. **(A)** Volcano plot comparing plasma protein levels between patients with detectable CDH11^+^ fibroblasts and those without (log₂ fold change vs. –log_10_p_adj_). Differentially expressed proteins (TIMP3, CXCL1 and ANGPT1) are highlighted in red color. Boxplots showing NPX values for these proteins in the two groups. Each point corresponds to an individual (light purple plot= patients without detectable CDH11+ fibroblasts, n= 13; dark purple plot= patients with detectable CDH11^+^ fibroblasts, n= 7). Horizontal line represents the median, while the whiskers extend to the 25th and 75th percentiles. Asterisks denote statistically significant differences between groups. **(B)** Pathway enrichment analysis of proteins elevated in patients with detectable CDH11^+^ fibroblasts. KEGG pathways (left) and Gene Ontology (GO) biological processes (right) are shown based on normalized enrichment scores. (CDH11: cadherin-11; TIMP3: tissue inhibitor of metalloproteinase 3; ANGPT1: angiopoietin 1; *, p_adj_< 0.050). **(C)** Representative confocal sections of RA patients’ synovial tissue (n=4; “pannus” acquired upon arthroplasty) depicting the expression of CD45 (green), CDH11 (red) and PDPN (magenta). The magnified panel shows one CDH11^+^CD45^+^ cell (leukocyte) being in close proximity to a CDH11^+^PDPN^+^CD45^-^ cell (fibroblast), as indicated by the white arrows. **(D)** Panel B accordingly depicts the expression of CD45 (green), CDH11 (red) and CD90 (magenta). The magnified panel shows one CDH11^+^CD45^+^ cell (leukocyte) being in close proximity to a CDH11^+^CD90^+^CD45^-^ cell (fibroblast), as indicated by the white arrow. **(E)** Panel C depicts the expression of Ly6G in green (surface marker protein for neutrophils), CDH11 (red) and CD90 (magenta). The magnified panel shows two neighboring cells, one CDH11^+^Ly6G^+^ cell (neutrophil) and one CDH11^+^CD90^+^ cell (fibroblast), as indicated by the white arrow. Scale bar is the same for all the images, as depicted in panel A. (CDH11: cadherin-11; PDPN: podoplanin; Ly6G: Lymphocyte antigen 6 complex locus G6D).

To gain further insights into the potential role of circulating CDH11^+^ fibroblasts, we evaluated the levels of proteins related to inflammation in matched plasma samples using the Olink technology platform. Of the 368 proteins measured with the Olink Explore 384 Inflammation panel, 358 (97%) passed stringent quality control and were included in the subsequent analyses. Comparison of the proteomic profiles between patients with detectable circulating CDH11^+^ fibroblasts (n=13) and those without (n=7) revealed significantly elevated levels of C-X-C Motif Chemokine Ligand 1 (CXCL1; p_adj_=0.039), angiopoietin 1 (ANGPT1; p_adj_= 0.039), and tissue inhibitor of metalloproteinases-3 (TIMP3; p_adj_= 0.039) in the former group (**Figure 6A**), proteins involved in angiogenesis, chemotaxis, and extracellular matrix remodeling. Moreover, pathway enrichment analysis indicated that the presence of circulating CDH11^+^ fibroblasts is associated with enhanced chemokine signaling (KEGG pathways) and increased cell-substrate adhesion (Gene Ontology biological processes), among other pathways. (**Figure 6B**).

In addition, we performed correlation analysis between individual circulating fibroblast, fibrocyte and CDH11^+^CD90^+^ neutrophil counts in all patients at baseline and plasma levels of proteins involved in the significantly enriched pathways. Circulating fibrocytes expressing CDH11 or CCR7 were positively correlated with IL-33 plasma levels, while forward correlations were also observed for CDH11^+^CD90^+^CCR6^+^ neutrophils with CCL28, Drebrin-like protein (DBNL), cytoplasmic protein NCK2, and breakpoint cluster region protein (BCR) (p_adj_<0.1; **Supplementary Table S9**), proteins involved in leukocyte chemotaxis, immune activation and cell signaling.

### 3.5 Rheumatoid synovium-infiltrating neutrophils may interact with synovial fibroblasts via CDH11

To investigate whether circulating leukocytes with mesenchymal phenotypic features could be found within synovial tissue and potentially interact with synovial fibroblasts, we conducted confocal microscopy analysis on synovial tissue biopsies obtained during knee arthroplasty from four patients with RA and three with osteoarthritis (OA). Using immunofluorescence staining for CD45, CDH11, PDPN, and CD90 on paraffin sections, the presence of CDH11^+^ leukocytes in close proximity to CDH11^+^ synovial fibroblasts was observed in RA (**Figure 6C**), indicating potential intercellular communication between these cells through homotypic binding. Moreover, co-expression of CD90 and CDH11 was detected on leukocytes and more specifically on neutrophils (Ly6G^+^) in RA, being closely located to CDH11^+^ fibroblasts (**Figures 6D, E**). Of note, none of these observations were evident in the OA synovium. Quantification across sections showed that CD90^+^CDH11^+^ neutrophils constituted a small subset, accounting for 4.2% (± 0.5%) of total Ly6G^+^ neutrophils, consistent with their low frequency in circulation. These findings suggest a neutrophil-fibroblast crosstalk mediated by CDH11, as well as a potential migratory pathway for the described cells from the periphery into the RA tissue, highlighting their possible relevance in the pathogenesis of inflammatory arthritis.

## 4 Discussion

Using mass cytometry, we show herein that numbers of circulating fibroblasts and fibrocytes, co-expressing CDH11 and chemokine receptors, possibly originating from inflamed joints and bone marrow, respectively, are increased in patients with RA and PsA. Mass cytometry is an ideal technology for identifying and characterizing rare single cells in whole blood samples (34, 35). Its minimal background noise and improved sensitivity make it particularly well-suited for the detection and characterization of low-abundance cellular events, such as circulating tumor cells in cancer patients, detected in frequencies ranging from 1 to 100 cells per ml of blood (36, 49).

Circulating fibroblasts and fibrocytes in our patients had a median cell count of 7 and 70 per ml of peripheral blood, respectively. Notably, the presence of circulating fibroblasts has been previously studied in RA (16), but not in PsA patients. Despite their low absolute numbers, these cells may be of major biological relevance, similar to circulating tumor cells which have been shown to impact clinical outcomes even at counts as low as 5 cells per 7.5 mL of blood (50, 51). Fibrocytes have been previously studied in RA by flow-cytometry, but again not in PsA, being elevated compared to controls at baseline, as well as during re-evaluation after three and six months (27). Increased fibrocyte frequencies have been reported in other inflammatory conditions (52–54), highlighting their association with systemic inflammation rather than disease specificity. Similarly, circulating fibroblasts, although not widely studied outside of rheumatic diseases, have been identified to be increased in conditions such as metastatic breast cancer (55). Therefore, the observed increase in circulating fibroblasts and fibrocytes in RA and PsA should not be interpreted as a disease-specific feature.

Circulating fibroblasts were primarily identified based on PDPN expression. Although TGF-β, IFN-γ, TNF-α, and IL-1 are known to shape the inflammatory environment and influence PDPN expression, our Olink analysis showed comparable levels of TGF-β, IFN-γ, TNF-α, IL-1α, and IL-1β across RA, PsA, and healthy controls (**Supplementary Figure S29**). These findings suggest that cytokine-driven modulation of PDPN expression likely had minimal impact on fibroblast detection in our study. By investigating the expression of markers that are known to characterize synovial fibroblasts, such as CDH11, CD90, CD34, and Notch3, we unveiled the heterogeneity of circulating fibroblasts, akin to their synovial counterparts (22). This heterogeneity was more evident in the circulating fibroblast pool of RA compared to PsA, suggesting potential differences in fibroblast involvement between the two types of arthritis, as also observed in synovium studies (32). Importantly, we acknowledge that not all fibroblast populations exert pro-inflammatory or pathogenic roles (56, 57). The complexity of fibroblast biology is further reflected in the altered composition of circulating fibroblast subsets observed in patients after three months of treatment, particularly among those achieving remission. These changes may indicate a therapeutic reshaping of the fibroblast compartment, mirroring the modulatory effects of therapy on these cells.

In both RA and PsA patients, circulating CD90^+^CDH11^+^ neutrophils were increased compared to controls, with their levels remaining elevated following three-month anti-rheumatic standard treatment, regardless of disease activity. The CD90 molecule, by binding to integrins, including αVβ3, is involved in the adhesion of leukocytes to activated human endothelial cells (EC), facilitating their extravasation into different tissues (47, 58, 59). Although certain studies have reported CDH11 and CD90 expression on immunocytes (17, 21, 47, 60), reports on direct CD90 expression on neutrophils are lacking. In our study, the first proteomic study to investigate the expression of these markers at single-cell level in the peripheral blood, the presence of leukocytes co-expressing CDH11 and CD90 was also detected in RA-derived synovial biopsies, but not in OA-derived, further supporting their potential migration into the rheumatoid synovium. These cells were located close to CDH11-expressing synovial fibroblasts, indicating their possible interaction through CDH11-mediated homotypic binding. In the context of RA, CD90 expression has been observed on CD14^+^ cells in perivascular areas of the synovium, possibly indicative of the migration of these cells from the peripheral blood to the synovial tissue (61). Moreover, mass cytometry data from synovial tissues has also revealed the expression of CDH11 and CD90 on certain monocytes, T and B cells (22). Interestingly, the levels of CDH11^+^CD90^+^ neutrophils that co-expressed Notch3 and CCR6 were also elevated in our patients, which may further support the migratory potential of this subset, as there is evidence suggesting the involvement of Notch3 in the process of immunocyte transmigration into the inflamed tissue, by mediating leukocyte adhesion to the endothelium (48). Although these findings are consistent with active trafficking of this neutrophil subset to inflamed joints, further studies are needed to determine whether these cells actively contribute to joint infiltration or instead reflect a systemically activated neutrophil state.

Circulating fibroblasts, fibrocytes and CDH11^+^CD90^+^ neutrophils mainly expressed CCR6, among the examined chemokine receptors CCR4, CCR7, CXCR3 and CXCR5. This may be indicative of their increased capacity to receive inflammatory signals from the joint, where its ligand, CCL20, is upregulated during arthritis (62). Moreover, increased counts of circulating fibroblasts and fibrocytes expressing CCR7 were observed in patients compared to controls. Although CCR7 is traditionally involved in the migration of cells toward lymphoid organs, its ligands CCL19 and CCL21 have been found to be highly produced in the synovium of both RA and PsA (63). Thus, CCR6 and CCR7 expressions may facilitate the guided movement of these cells from the bloodstream toward distant joints.

Patients with circulating CDH11^+^ fibroblasts in their blood had elevated CXCL1, ANGPT1 and TIMP3 levels in their matched plasma samples. CXCL1 is a potent neutrophil chemoattractant that is notably overexpressed in the inflamed synovium (64). Interestingly, engagement of CDH11 on synovial fibroblasts has been shown to increase CXCL1 secretion by these cells (65). ANGPT1 and TIMP3, on the other hand, are involved in vascular stabilization (66) and inhibition of matrix-metalloproteinases (67), respectively. The elevated levels of these molecules may therefore reflect a compensatory systemic response aimed at counteracting inflammation. Moreover, the association between the presence of circulating fibroblasts and the enrichment of pathways related to chemokine signaling and cell adhesion further supports the hypothesis that CDH11^+^ fibroblasts may possess enhanced migratory and tissue-invasive capabilities.

Circulating fibrocyte counts were found to positively correlate with IL-33, an alarmin cytokine known to promote cell activation and proliferation as a response to inflammatory stimuli (68), which has been implicated in the modulation of fibrocyte function (68, 69). Furthermore, positive correlations between CDH11^+^CD90^+^CCR6^+^ neutrophil counts and CCL28, DBNL, NCK2 and BCR plasma levels suggest an association of this neutrophil subset with a pro-migratory and immunologically active plasma environment (70–74). Together, these findings highlight a potential mechanistic link between specific cytokines and signaling pathways and the migratory behavior of the identified circulating fibrocytes and neutrophils.

The concept of circulating synovial fibroblasts was initially proposed as a hypothesis to explain inflammatory monoarthritis/oligoarthritis progression to polyarthritis, with studies showing the ability of injected pathogenic fibroblasts to a joint to cause arthritis and then migrate to unaffected joints, where they undergo proliferation and promote a pro-inflammatory microenvironment (13). Given that the mesenchymal CDH11, which is essential for the development of the synovium (75), has been previously detected in RA patients’ peripheral blood (17) and is also a marker gene for circulating PRIME cells (16), it can be hypothesized that specific fibroblast subpopulations are activated and migrate into the bloodstream toward distant joints, thus spreading arthritis. Nevertheless, our data derives from patients with established disease and limited longitudinal sampling, therefore we cannot address whether the presence of these circulating populations represents an early, precursor event or is instead a downstream consequence of sustained inflammation.

**Figure 7:**
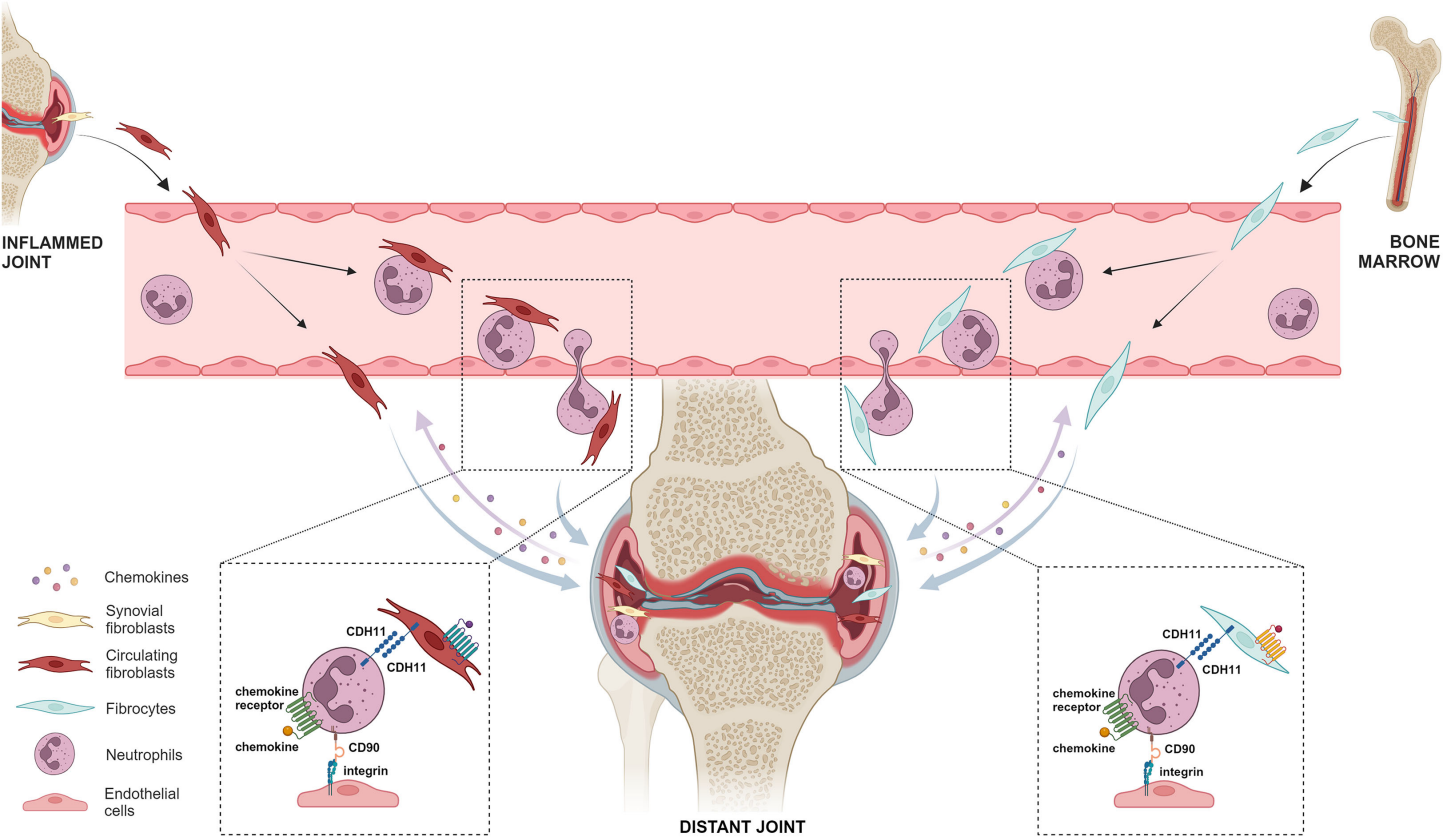
Proposed fibroblast and fibrocyte migration model through the circulation in inflammatory arthritis. Circulating fibroblasts and fibrocytes, originating from inflamed joints and bone marrow, respectively, may migrate into the distant synovium, either alone or by forming complexes with CDH11^+^CD90^+^ neutrophils, through CDH11-mediated homotypic binding. This process may be facilitated by a) binding of CD90 to integrins of endothelial cells during extravasation, and b) cell attraction by inflamed synovium-derived chemokines. This model may operate in RA, as well as in PsA. Created with BioRender.com. (CDH11, cadherin-11).

Building upon the findings of the aforementioned studies (13, 15–17) and based on the results presented herein, we propose a model where fibroblasts and fibrocytes, expressing CDH11, originating from the joints and bone marrow, respectively, enter the bloodstream and migrate into distant synovia, either alone or by forming complexes with CDH11^+^CD90^+^ neutrophils (**Figure 7**). Such migration processes may operate independently of adaptive immunity-mediated pathogenetic mechanisms, which is in line with our mass cytometry findings. According to our proposed model, migration of fibroblasts and fibrocytes along with neutrophils may be facilitated by binding of CD90 to integrins of endothelial cells during extravasation, with these cells being attracted towards the joint by synovium-derived chemokines. Homotypic binding via CDH11 may enable the formation of cell clusters with enhanced migratory potential that could possibly insert into the joint. As in the case of circulating tumor cell clusters, where non–tumor cells, such as platelets and neutrophils, can interact with tumor cells to enhance their survival and metastasis (76, 77), we could assume that the formation of cell aggregates between circulating fibroblasts/fibrocytes and neutrophils may protect these rare circulating cells against harmful shear stress and mechanical forces formed in the bloodstream. Moreover, PDPN on fibroblasts may bind to C‐type lectin‐like receptor 2, which is mainly expressed by platelets, potentially resulting in platelet activation (78) which is known to support tumor dissemination (78, 79). Therefore, circulating fibroblasts may bind to neutrophils and platelets via PDPN, further facilitating their trafficking through circulation. Moreover, since the levels of circulating CDH11-expressing fibroblasts, fibrocytes and neutrophils remain elevated regardless of the disease activity of our patients, the proposed model seems to not being fully affected by current DMARD-based therapies. Larger longitudinal studies with treatment-stratified analyses are needed to determine whether specific therapies reduce these populations over time and therefore support the endeavors for the development of anti-mesenchymal therapies, targeting, for example, CDH11 (80, 81).

The major limitation of our study is that we have not explored potential functional implications associated with the rare cell populations identified in the peripheral blood. *In vivo* and *ex vivo* experiments, including xenotransplantation of patient-derived circulating fibroblasts in SCID mice, similar to previous studies (15), could provide stronger evidence to further support the origin, homing and pathogenic potential of the identified circulating fibroblasts. Functional *vitro* assays to directly evaluate the inflammatory capacity of the identified subsets as well as clinical correlations in a wider cohort could also provide deeper insight into their pathogenetic role and potential as therapeutic targets. However, these assays require approximately 10,000 sorted circulating CDH11^+^ fibroblasts, a number that cannot be feasibly obtained from peripheral blood. Technologies used for rare circulating tumor cells, such as Parsortix^®^ or CellSearch^®^ (77, 82), could overcome this constraint, but they are not currently applicable to fibroblasts. We also acknowledge the lack of orthogonal validation of the presence of circulating fibroblasts using independent approaches (e.g., scRNA-seq), which was constrained by the absence of suitable publicly available peripheral blood single-cell datasets with sufficient resolution to enable direct comparison. Technical aspects also need to be considered, as cell recovery in mass cytometry is always less than 100%, and the adhesive properties of CDH11 may exclude clusters of positive cells from single-cell analysis, suggesting that actual numbers could be higher than observed. In addition, paired bone marrow sampling was available only for two PsA patients with concomitant hematologic abnormalities, therefore conclusions regarding fibroblast/fibrocyte origin should be interpreted cautiously and require further confirmation in larger cohorts using complementary approaches, such as clonal tracking or paired transcriptomic comparisons. Finally, confocal microscopy was performed in archival tissues, so direct paired analysis between peripheral blood and tissue samples was not possible.

To conclude, our results form the basis for future experiments to extend our understanding of the pathogenetic role of circulating cells with mesenchymal phenotype, forming cell-cell complexes, and the dynamic cellular interactions leading to arthritis ‘spreading’ or “metastasis”. Based on previous studies (13, 15–17) and our findings, arthritis may be spread by a process shared by RA and PsA, which operates independently of adaptive immunity and current drugs. Thus, further studies may reveal new, fibroblast-associated targets for therapeutic intervention in chronic inflammatory arthritis.

## Data Availability

The original contributions presented in the study are included in the article/**Supplementary Material**. Further inquiries can be directed to the corresponding author.
