# Supplementary material for: Circulating fibroblasts and neutrophils co-expressing CDH11+ and chemokine receptors in rheumatoid and psoriatic arthritis: a shared mechanism of ‘arthritis spreading’?
Source: Front Immunol. 2026 Jan 16;16:1743919. doi: 10.3389/fimmu.2025.1743919 (PMC12855083; doi:10.3389/fimmu.2025.1743919)
Supplement: Supplementary file 1 [file DataSheet1.pdf]

Supplementary Material

Circulating fibroblasts and neutrophils co-expressing CDH11$^+$ and chemokine receptors in patients with rheumatoid and psoriatic arthritis: a shared mechanism of ‘arthritis spreading ‘?

Maria Kyriakidi[#], Eleni-Kyriaki Vetsika[#*], George E Fragoulis, Maria Sakkou, Kleio-Maria Verrou, Anastasios Mourikis, Nikolaos I Vlachogiannis, Maria G Tektonidou, Petros P Sfikakis

[#] These authors contributed equally to this work

*** Correspondence:** Eleni-Kyriaki Vetsika: ekvetsika@med.uoa.gr

1 Supplementary Figures and Tables

1.1 Supplementary Figures

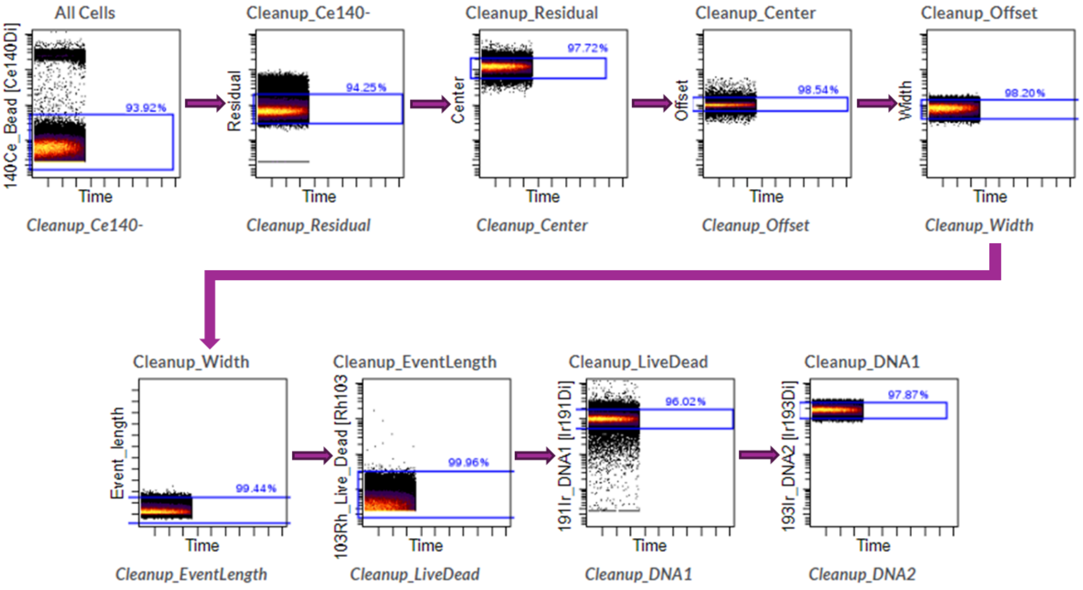

Supplementary Figure S1. Representative dot plots showing the gating strategy for data cleanup to remove aggregates, debris, normalization beads, doublets, and dead cells from further analysis. Arrows indicate the gating sequence. Percentages shown in the plots represent the frequency of each gated population relative to its immediate parent population, as defined by the population name listed at the top of each plot.

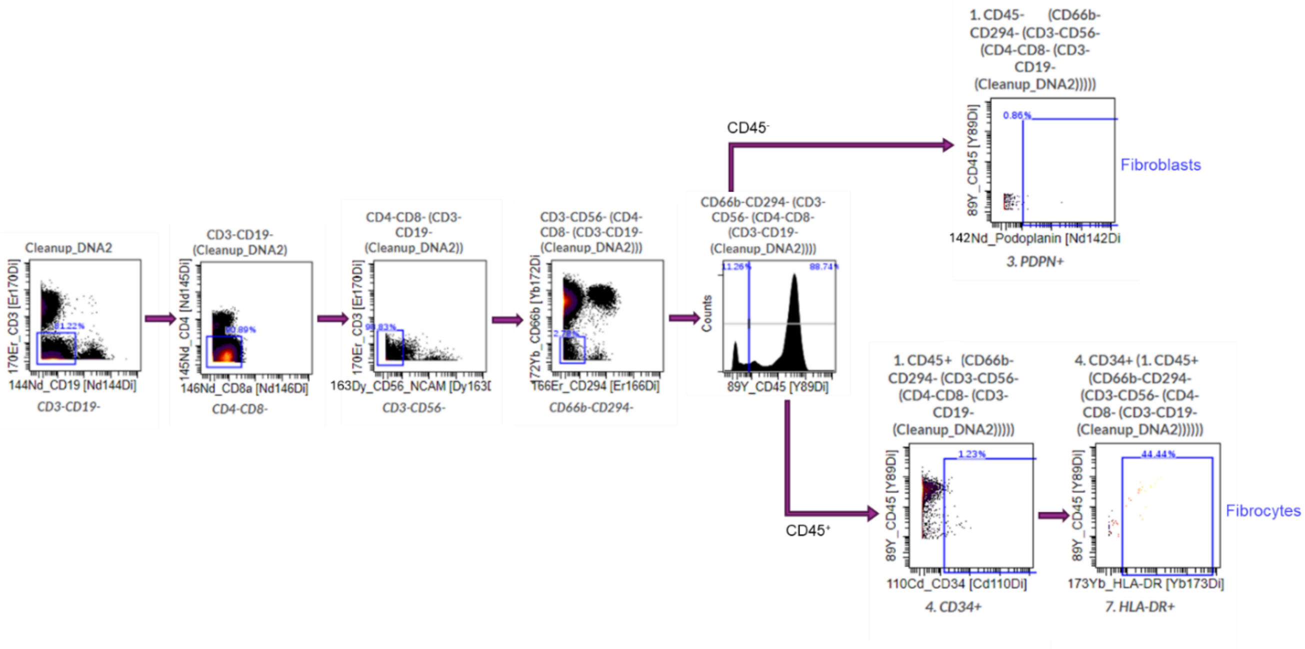

Supplementary Figure S2. Representative dot plots showing the gating strategy for the identification of circulating fibroblasts and fibrocytes. Arrows indicate the gating sequence. Percentages shown in the plots represent the frequency of each gated population relative to its immediate parent population, as defined by the population name listed at the top of each plot.

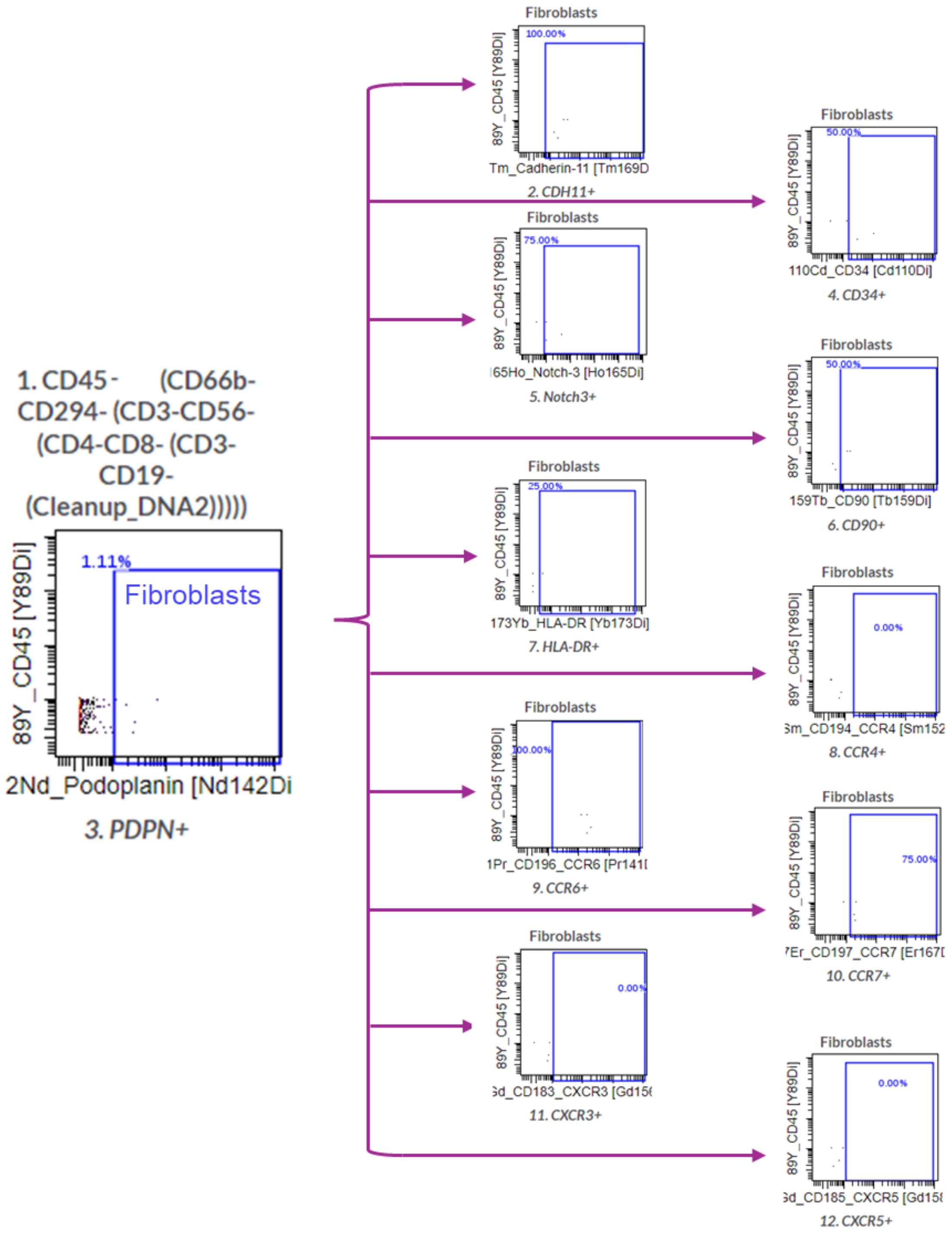

Supplementary Figure S3. Representative dot plots showing the gating strategy for the phenotypic analysis of CDH11^{+}, CD34^{+}, Notch3^{+}, CD90^{+}, HLA-DR^{+}, CCR4^{+}, CCR6^{+}, CCR7^{+}, CXCR3^{+} and CXCR5^{+} circulating fibroblasts. Arrows indicate the gating sequence. Percentages shown in the plots represent the frequency of each gated population relative to its immediate parent population, as defined by the population name listed at the top of each plot.

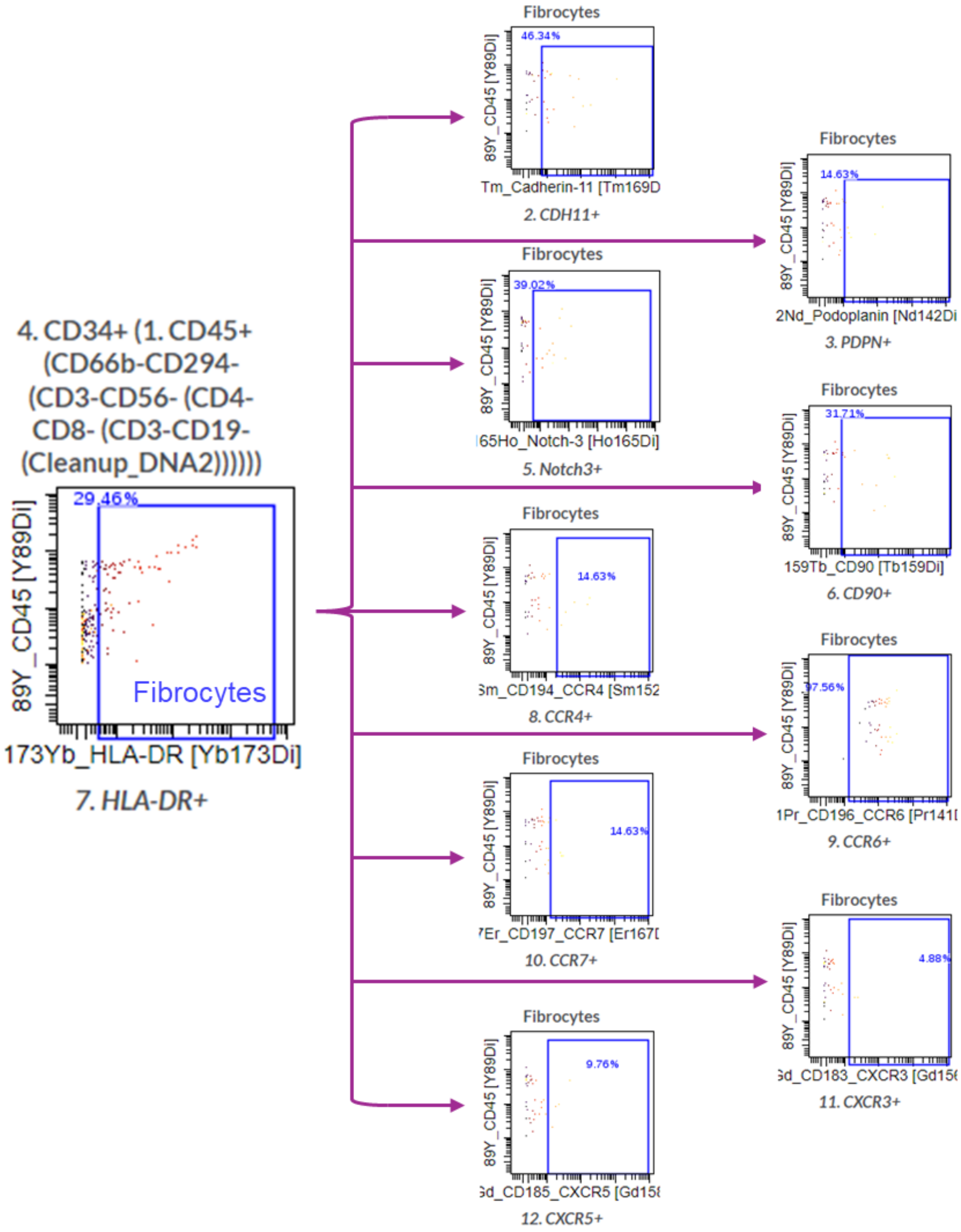

Supplementary Figure S4. Representative dot plots showing the gating strategy for the phenotypic analysis of CDH11$^+$, PDPN$^+$, Notch3$^+$, CD90$^+$, CCR4$^+$, CCR6$^+$, CCR7$^+$, CXCR3$^+$ and CXCR5$^+$ circulating fibrocytes. Arrows indicate the gating sequence. Percentages shown in the plots represent the frequency of each gated population relative to its immediate parent population, as defined by the population name listed at the top of each plot.

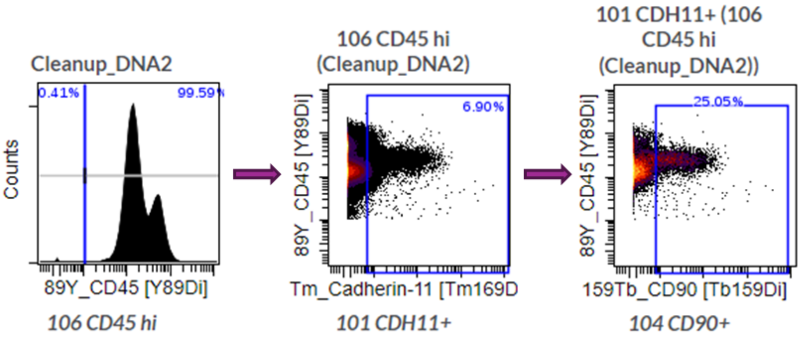

Supplementary Figure S5. Representative dot plots showing the gating strategy for the phenotypic analysis of CD90^{+}CDH11^{+} CD45^{+} cells. Arrows indicate the gating sequence. Percentages shown in the plots represent the frequency of each gated population relative to its immediate parent population, as defined by the population name listed at the top of each plot.

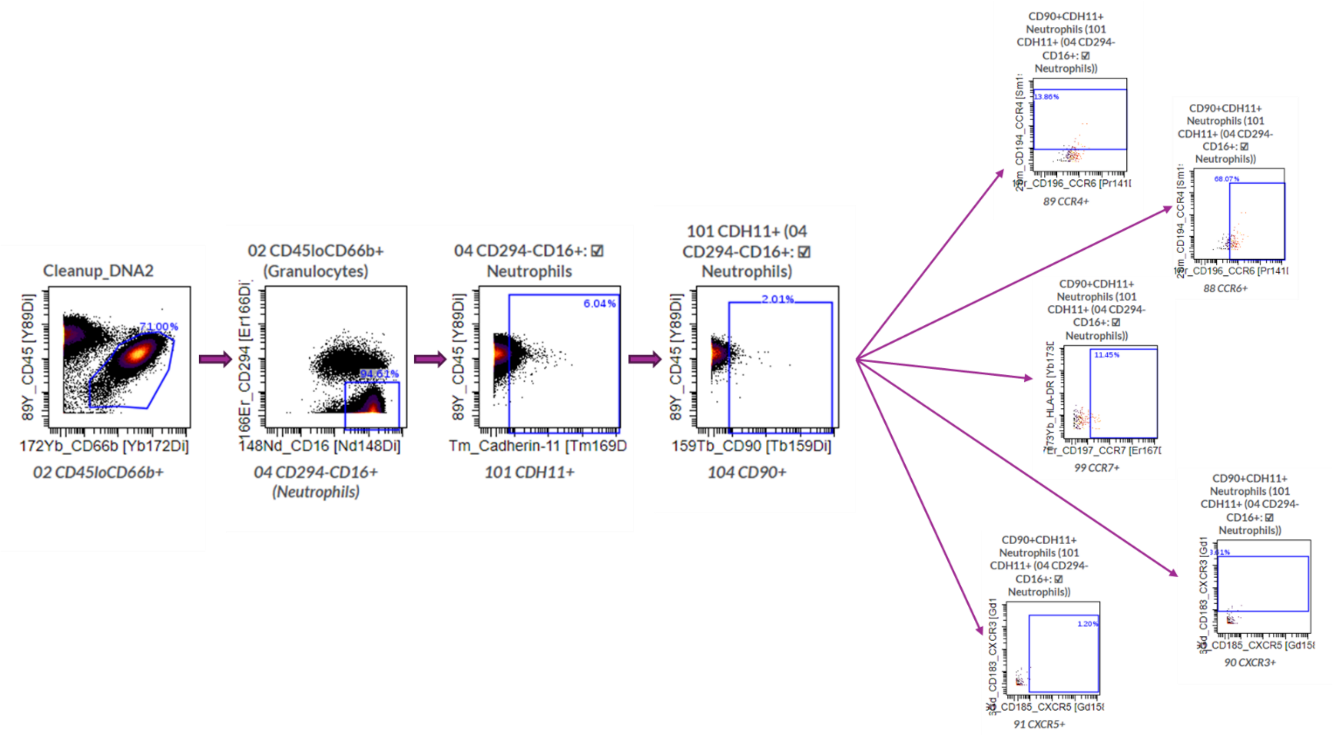

Supplementary Figure S6. Representative dot plots showing the gating strategy for the phenotypic analysis of total CD90^{+}CDH11^{+}neutrophils as well as CCR4^{+}, CCR6^{+}, CCR7^{+}, CXCR3^{+}and CXCR5^{+} CD90^{+}CDH11^{+} neutrophils. Arrows indicate the gating sequence. Percentages shown in the plots represent the frequency of each gated population relative to its immediate parent population, as defined by the population name listed at the top of each plot.

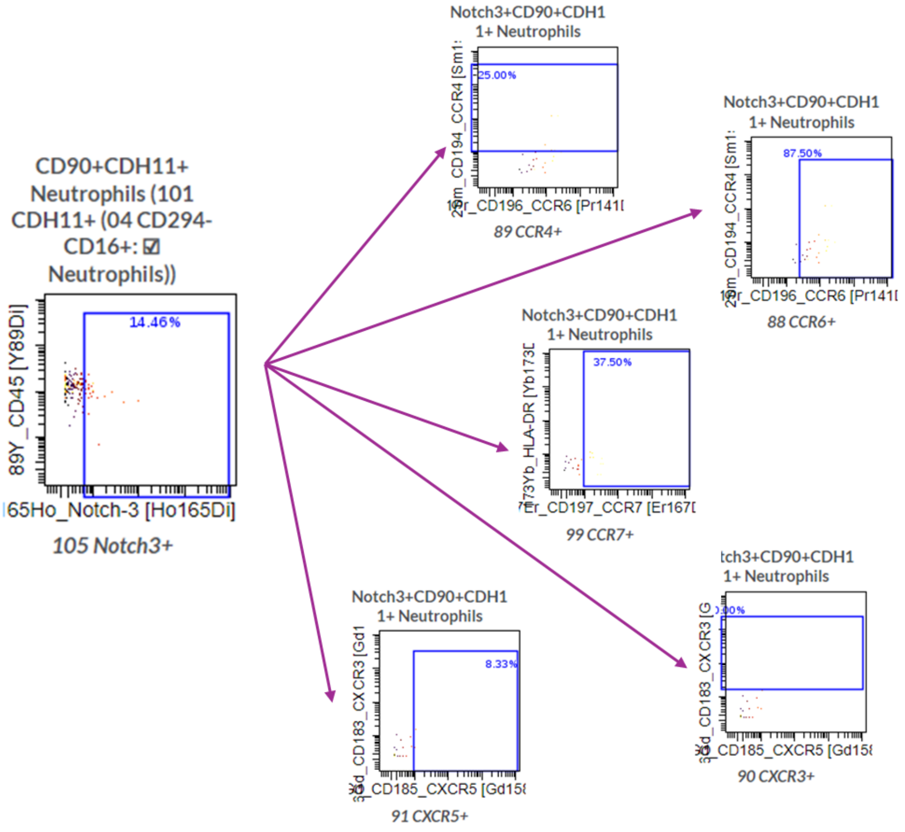

Supplementary Figure S7. Representative dot plots showing the gating strategy for the phenotypic analysis of total Notch3^{+}CD90^{+}CDH11^{+} neutrophils as well as CCR4^{+}, CCR6^{+}, CCR7^{+}, CXCR3^{+} and CXCR5^{+} Notch3^{+}CD90^{+}CDH11^{+} neutrophils. Arrows indicate the gating sequence. Percentages shown in the plots represent the frequency of each gated population relative to its immediate parent population, as defined by the population name listed at the top of each plot.

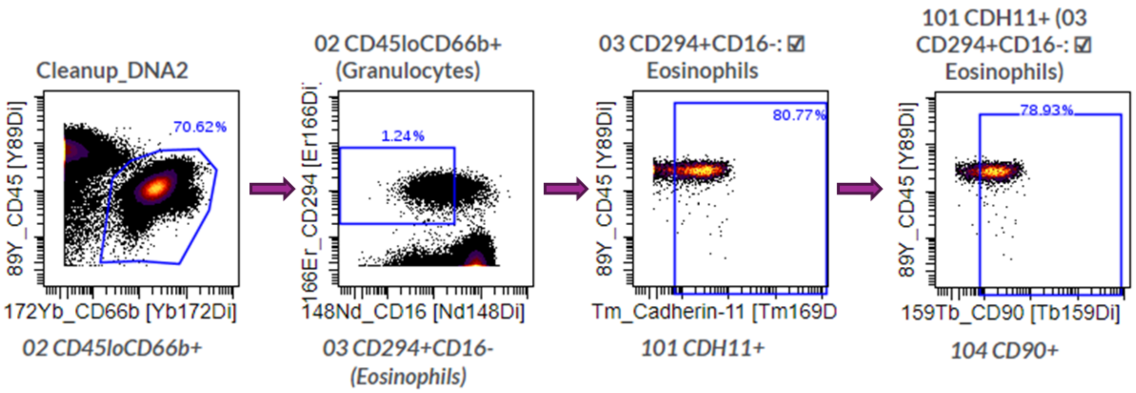

Supplementary Figure S8. Representative dot plots showing the gating strategy for the phenotypic analysis of CD90^{+}CDH11^{+} eosinophils. Arrows indicate the gating sequence.

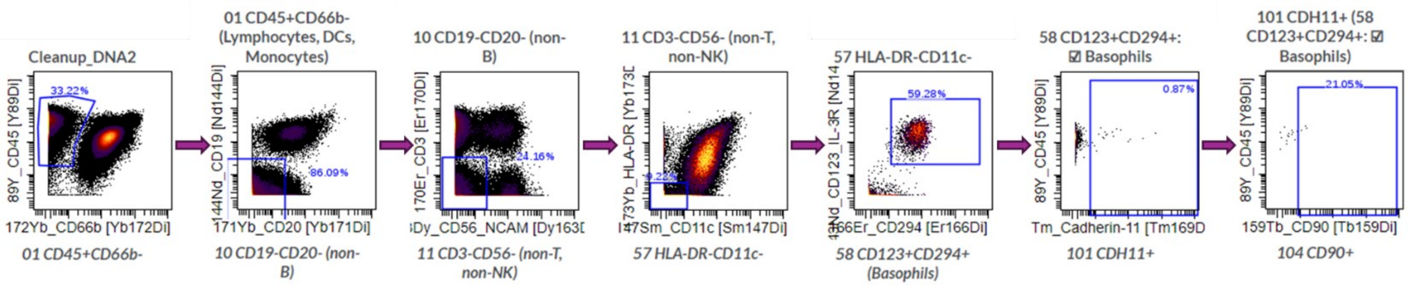

Supplementary Figure S9. Representative dot plots showing the gating strategy for the phenotypic analysis of CD90^{+}CDH11^{+} basophils. Arrows indicate the gating sequence. Percentages shown in the plots represent the frequency of each gated population relative to its immediate parent population, as defined by the population name listed at the top of each plot.

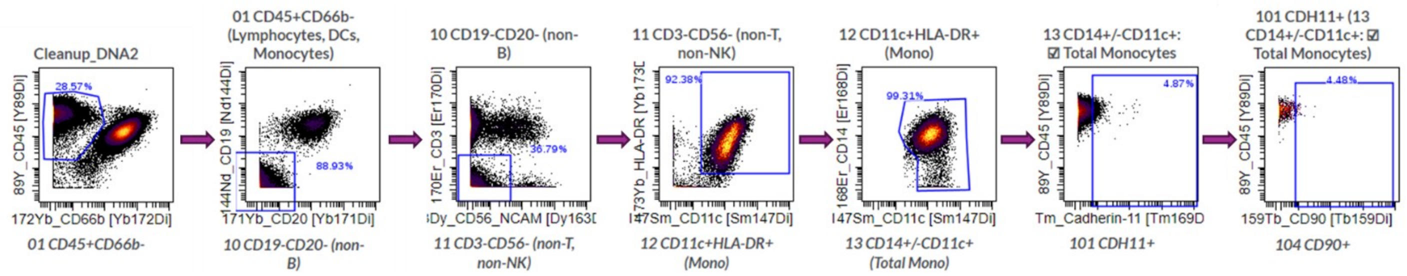

Supplementary Figure S10. Representative dot plots showing the gating strategy for the phenotypic analysis of CD90^{+}CDH11^{+} monocytes. Arrows indicate the gating sequence. Percentages shown in the plots represent the frequency of each gated population relative to its immediate parent population, as defined by the population name listed at the top of each plot.

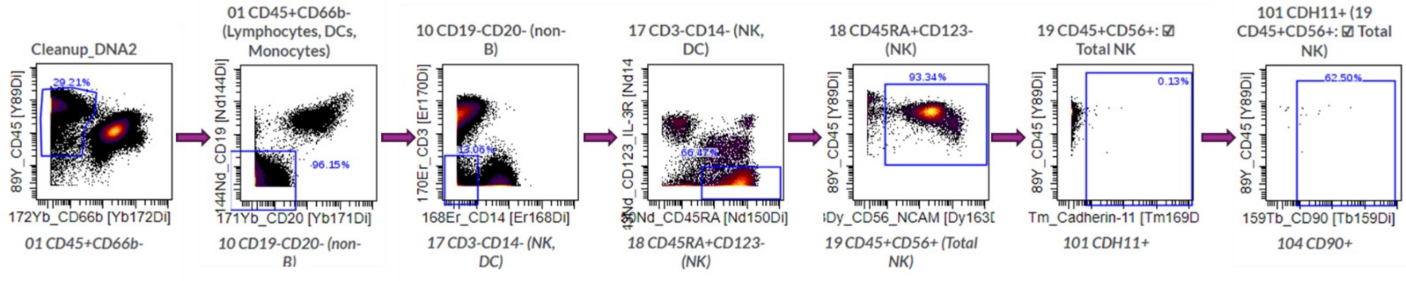

Supplementary Figure S11. Representative dot plots showing the gating strategy for the phenotypic analysis of CD90^{+}CDH11^{+} NK cells. Arrows indicate the gating sequence. Percentages shown in the plots represent the frequency of each gated population relative to its immediate parent population, as defined by the population name listed at the top of each plot. (NK: natural killer)

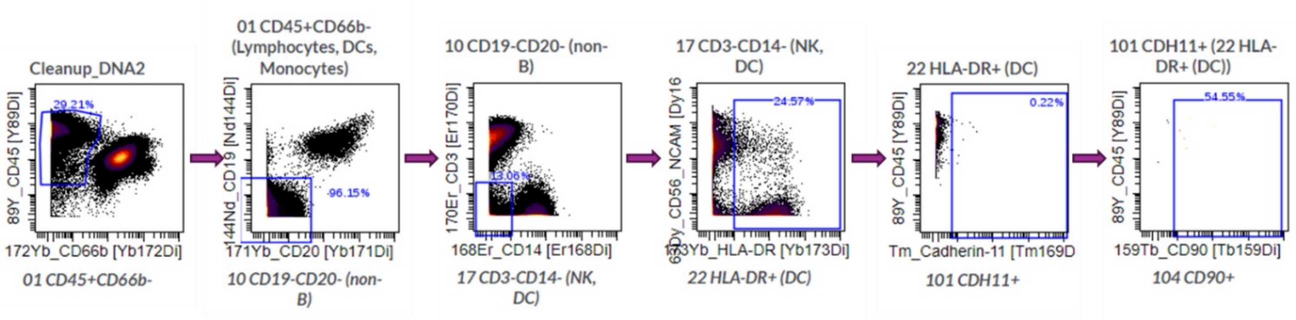

Supplementary Figure S12. Representative dot plots showing the gating strategy for the phenotypic analysis of $CD90^{+}CDH11^{+}$ DC. Arrows indicate the gating sequence. Percentages shown in the plots represent the frequency of each gated population relative to its immediate parent population, as defined by the population name listed at the top of each plot. (DC: dendritic cells)

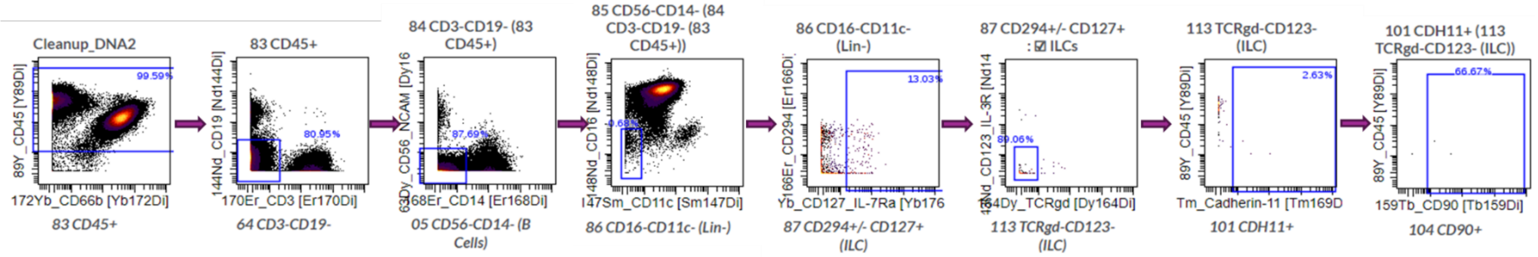

Supplementary Figure S13. Representative dot plots showing the gating strategy for the phenotypic analysis of $CD90^{+}CDH11^{+}$ ILC. Arrows indicate the gating sequence. Percentages shown in the plots represent the frequency of each gated population relative to its immediate parent population, as defined by the population name listed at the top of each plot. (ILC: innate lymphoid cells)

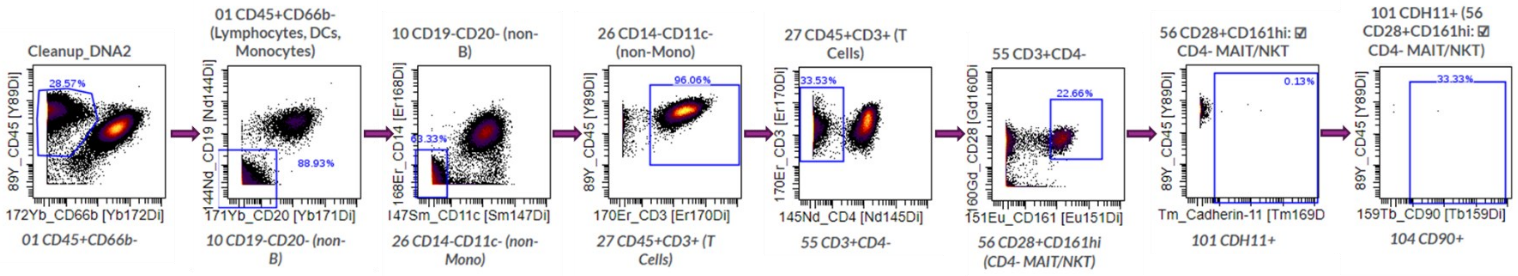

Supplementary Figure S14. Representative dot plots showing the gating strategy for the phenotypic analysis of $CD90^{+}CDH11^{+}$ MAIT/iNKT. Arrows indicate the gating sequence. Percentages shown in the plots represent the frequency of each gated population relative to its immediate parent population, as defined by the population name listed at the top of each plot. (MAIT: mucosal-associated invariant T cells; iNKT: invariant natural killer T)

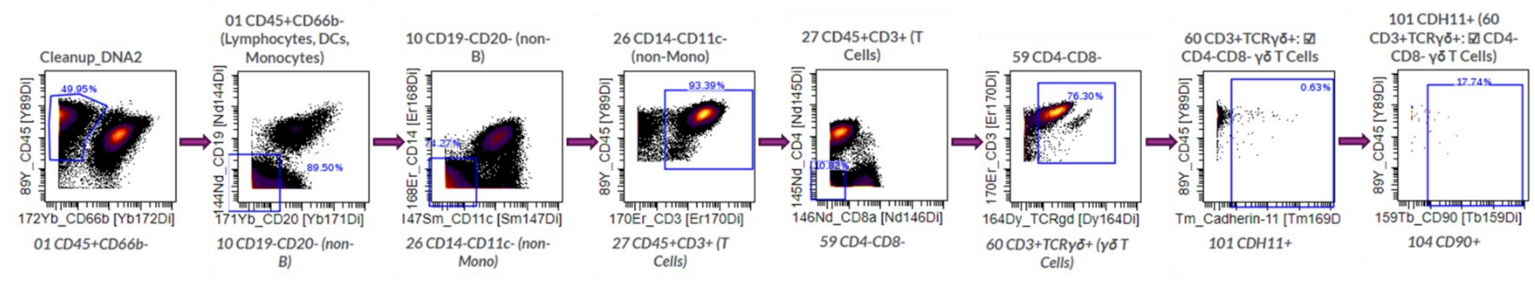

Supplementary Figure S15. Representative dot plots showing the gating strategy for the phenotypic analysis of CD90^{+}CDH11^{+} γδ T cells. Arrows indicate the gating sequence. Percentages shown in the plots represent the frequency of each gated population relative to its immediate parent population, as defined by the population name listed at the top of each plot.

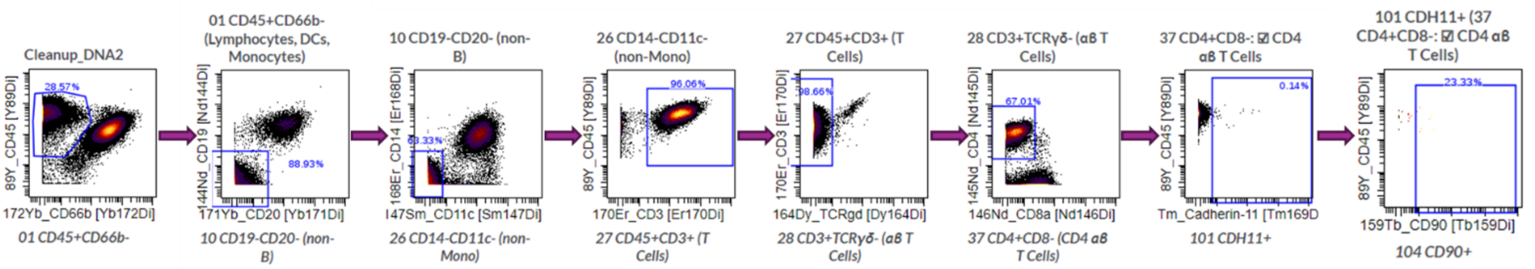

Supplementary Figure S16. Representative dot plots showing the gating strategy for the phenotypic analysis of CD90^{+}CDH11^{+} CD4^{+} T cells. Arrows indicate the gating sequence. Percentages shown in the plots represent the frequency of each gated population relative to its immediate parent population, as defined by the population name listed at the top of each plot.

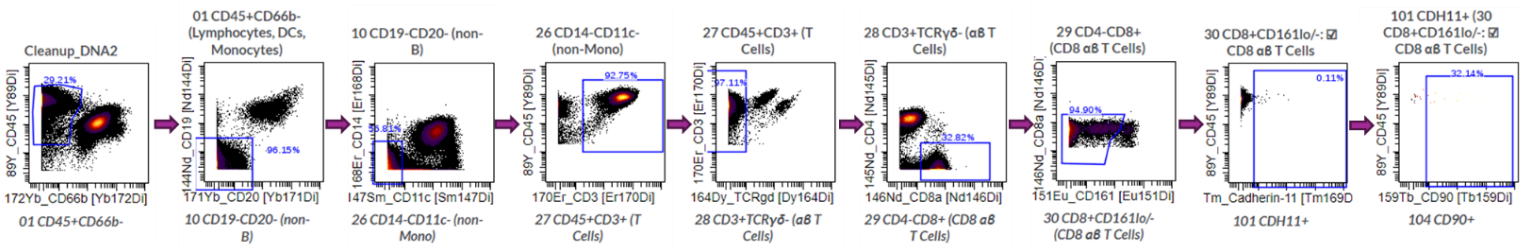

Supplementary Figure S17. Representative dot plots showing the gating strategy for the phenotypic analysis of CD90^{+}CDH11^{+} CD8^{+} T cells. Arrows indicate the gating sequence. Percentages shown in the plots represent the frequency of each gated population relative to its immediate parent population, as defined by the population name listed at the top of each plot.

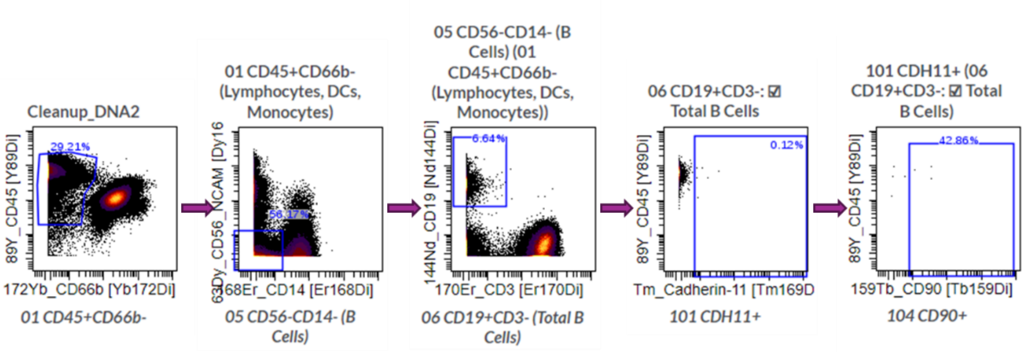

Supplementary Figure S18. Representative dot plots showing the gating strategy for the phenotypic analysis of $CD90^{+}CDH11^{+}$ B cells. Arrows indicate the gating sequence. Percentages shown in the plots represent the frequency of each gated population relative to its immediate parent population, as defined by the population name listed at the top of each plot.

A

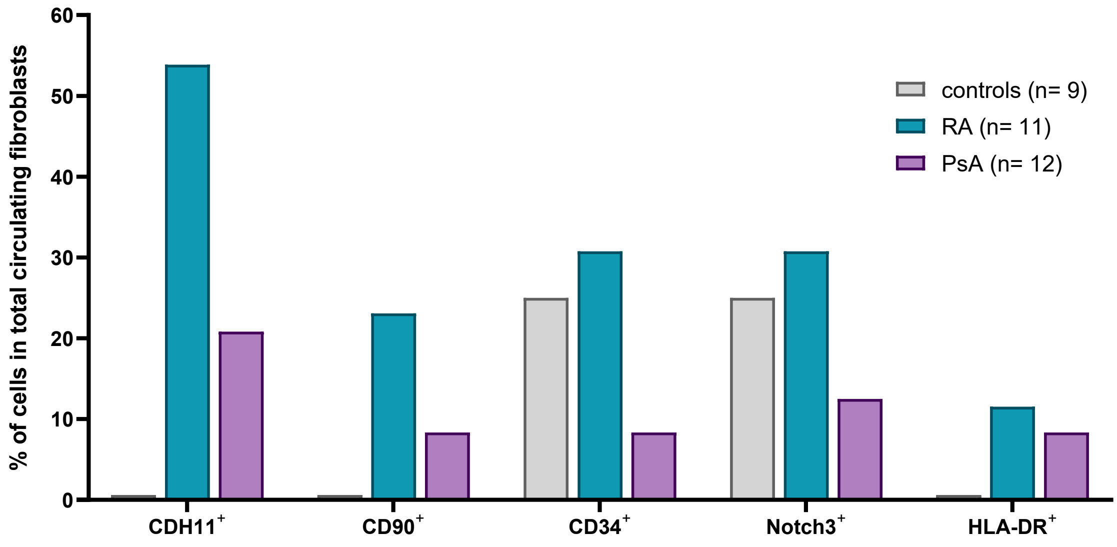

B

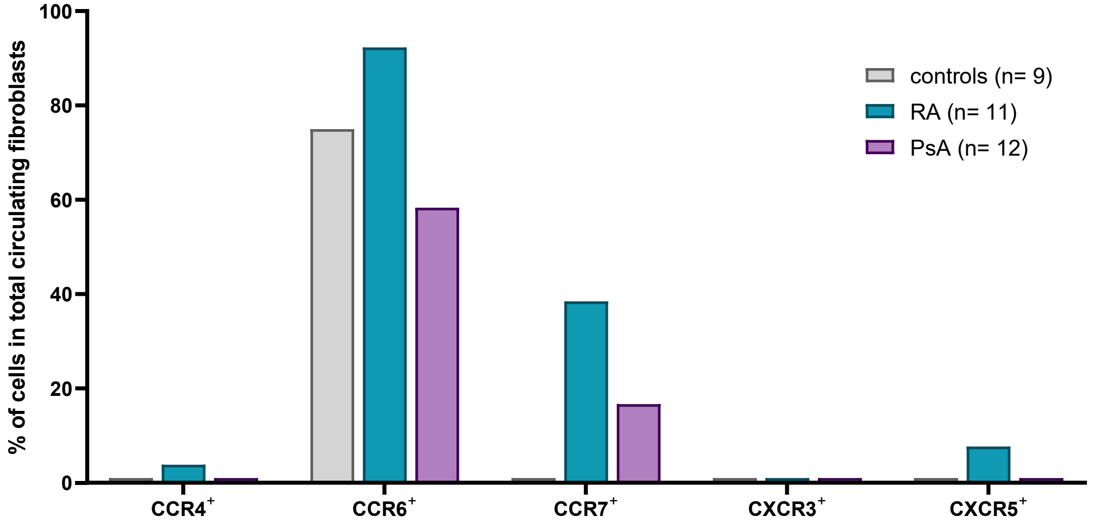

Supplementary Figure S19. Phenotypic characterization of circulating fibroblasts in patients and controls. (**A**) Bar plots depicting the percentages of CDH11$^+$, CD90$^+$, CD34$^+$, Notch3$^+$ and HLA-DR$^+$ circulating fibroblasts within total detected fibroblasts per study group. (**B**) Bar plots showing the percentages of CCR4$^+$, CCR6$^+$, CCR7$^+$, CXCR3$^+$ and CXCR5$^+$ circulating fibroblasts within total detected fibroblasts per study group (grey plot= controls, n= 9; cyan plot= RA, n= 11; purple plot= PsA, n= 12). Percentages are calculated by aggregating cell counts across all individuals of each study group. (n: number of individuals; RA: rheumatoid arthritis; PsA: psoriatic arthritis)

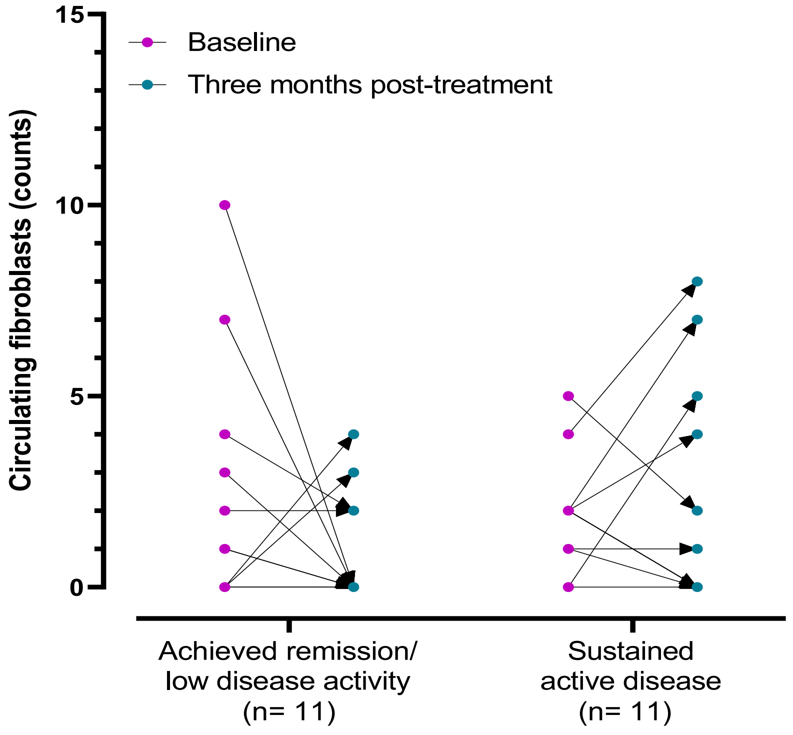

Supplementary Figure S20. Fibroblast counts measured three-months post-treatment are comparable to baseline numbers. Total fibroblast counts in patients who achieved remission (n= 11) or remained clinically active (n= 11) post-treatment, measured at baseline and in their three-month follow-up. Each point corresponds to an individual patient (magenta points= baseline levels; cyan points= three months post-treatment levels). Comparison of paired samples between timepoints was performed using Wilcoxon test. (n: number of individuals)

A

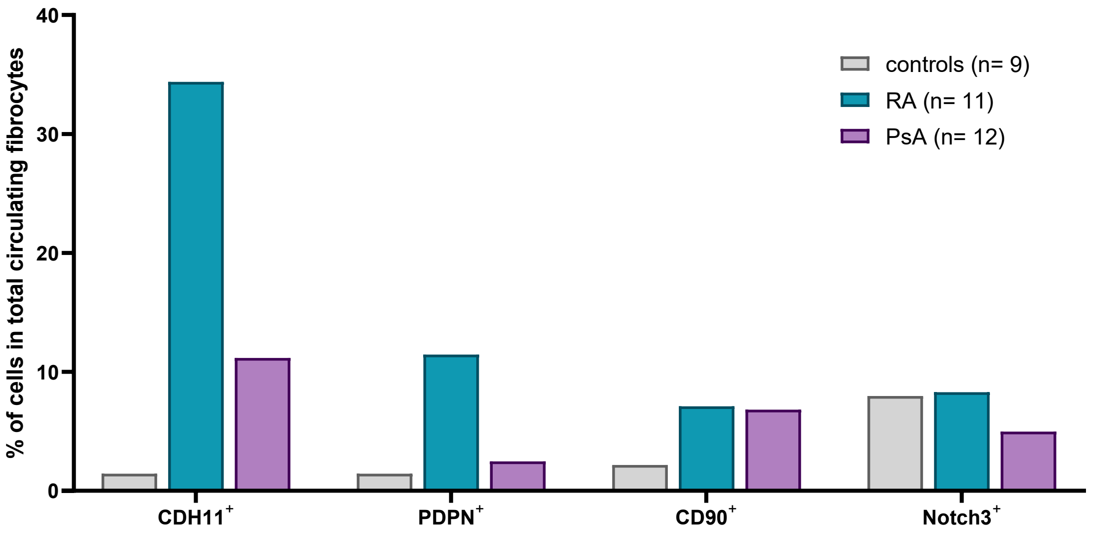

B

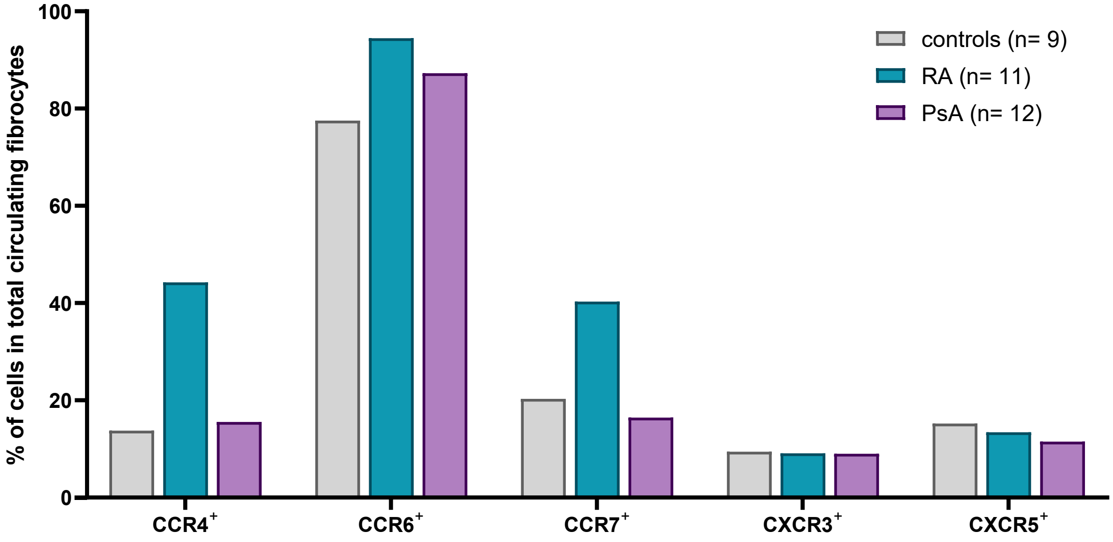

Supplementary Figure S21. Phenotypic characterization of circulating fibrocytes in patients and controls. (**A**) Bar plots depicting the percentages of CDH11$^+$, PDPN$^+$, CD34$^+$ and Notch3$^+$ fibrocytes within total detected fibroblasts per study group. (**B**) Bar plots showing the percentages of CCR4$^+$, CCR6$^+$, CCR7$^+$, CXCR3$^+$ and CXCR5$^+$ fibroblasts within total detected fibroblasts per study group (grey plot= controls, n= 9; cyan plot= RA, n= 11; purple plot= PsA, n= 12). Percentages are calculated by aggregating cell counts across all individuals of each study group. (n: number of individuals; RA: rheumatoid arthritis; PsA: psoriatic arthritis)

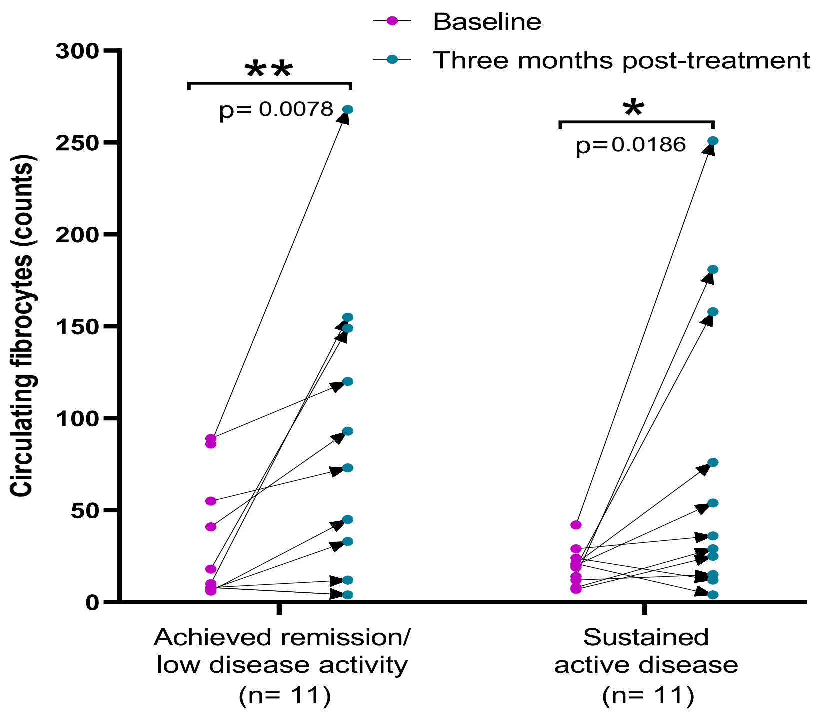

Supplementary Figure S22. Fibrocyte counts are elevated three months post-treatment, regardless of patients' clinical status. Total fibrocyte counts in patients who became inactive (n= 11) or remained active (n= 11) post-treatment, measured at baseline and in their three-month follow-up. Each point corresponds to an individual patient (magenta points= baseline levels; cyan points= three months post-treatment levels). Comparison of paired samples between timepoints was performed using Wilcoxon test. Asterisks denote statistically significant differences between timepoints. (n: number of individuals; *, $p< 0.050$; **, $p\leq 0.010$)

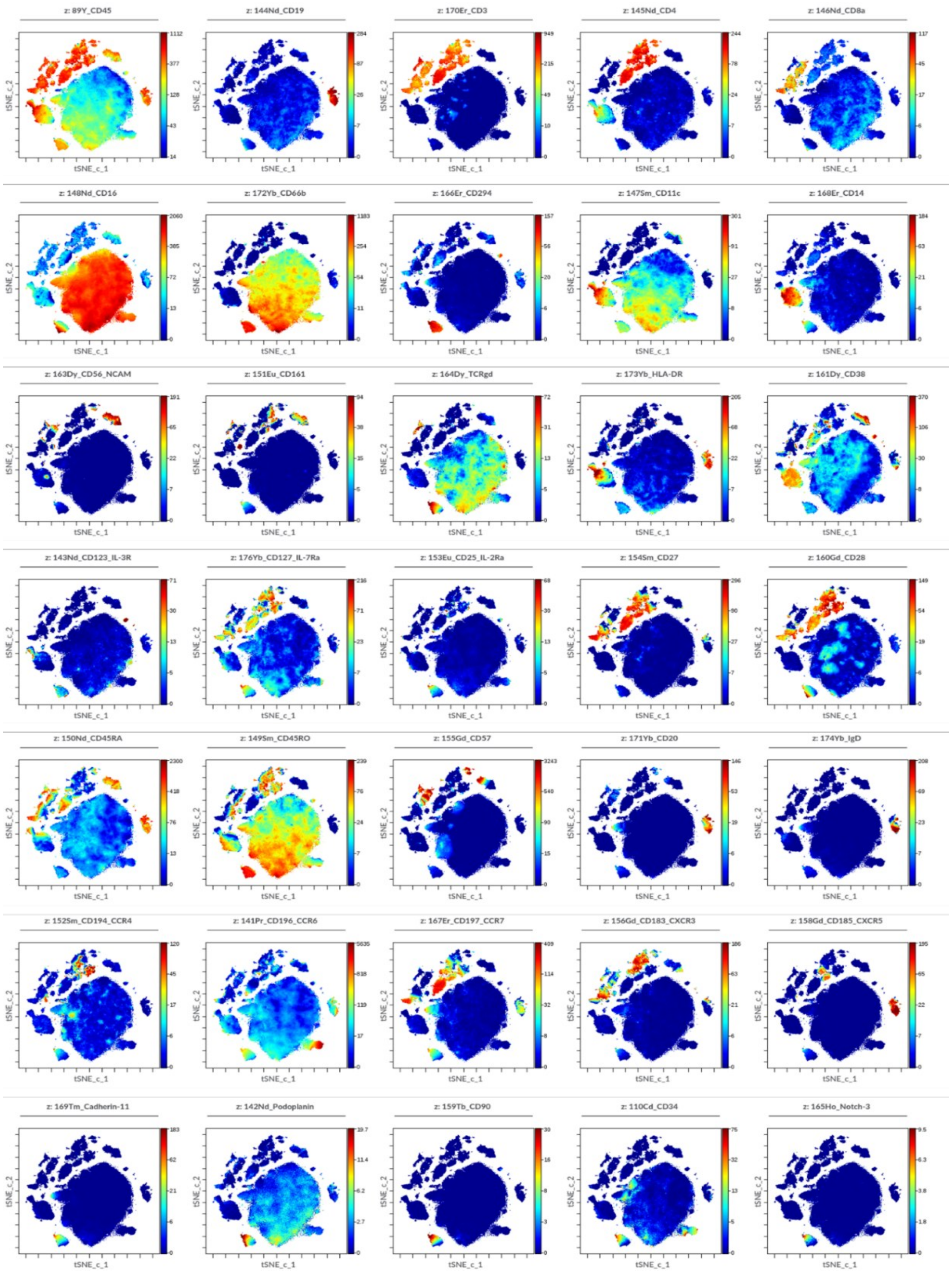

Supplementary Figure S23. tSNE-CUDA plots showing the expression of the 36 markers used on the CyTOF analysis on 810,000 randomly selected cells. CD45, CD19, CD3, CD4, CD8a, CD16, CD66b, CD294, CD11c, CD14, CD56, CD161, TCRgd, HLA-DR, CD38, CD123/IL-3R, CD127/IL-7Ra, CD25/IL-2Ra, CD27, CD28, CD45RA, CD45RO, CD57, CD20, IgD, CD194/CCR4, CD196/CCR6, CD197/CCR7, CD183/CXCR3, CD185/CXCR5, CDH11, PDPN, CD90, CD34, Notch3 are plotted separately in the 2-dimensional space. Each dot represents a cell and is colored according to marker's intensity on a spectrum heat scale (red= high intensity; blue=low intensity). Arcsine-transformed color scales report the raw values of the marker's intensity.

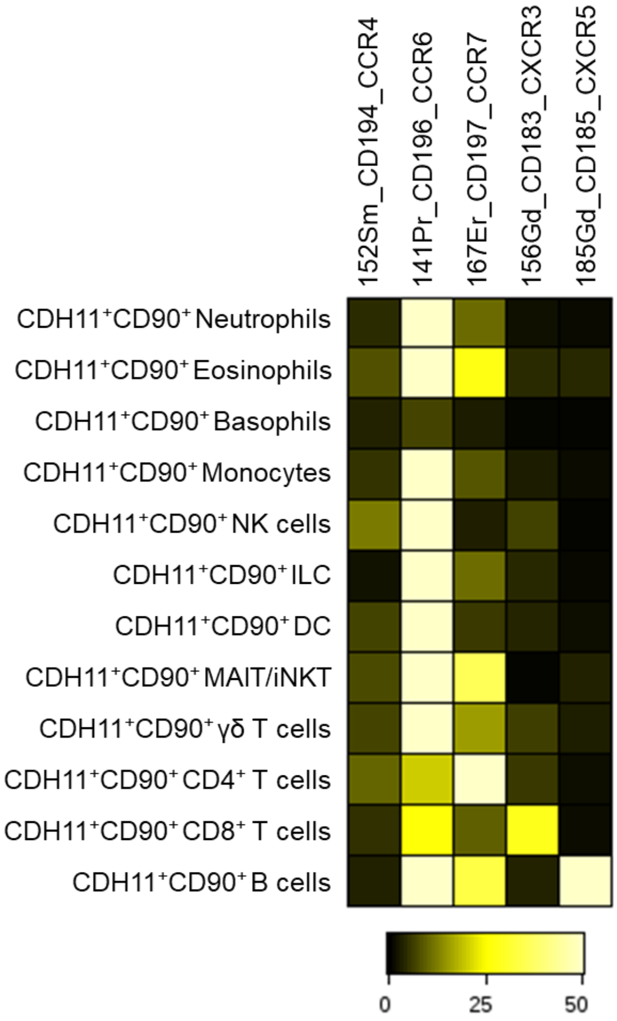

Supplementary Figure S24. Expression of chemokine receptors by circulating leukocyte subpopulations co-expressing CDH11 and CD90. Heatmap showing the expression levels (median intensity) of CCR4, CCR6, CCR7, CXCR3 and CXCR5 within each CDH11$^+$CD90$^+$ leukocyte subpopulation.

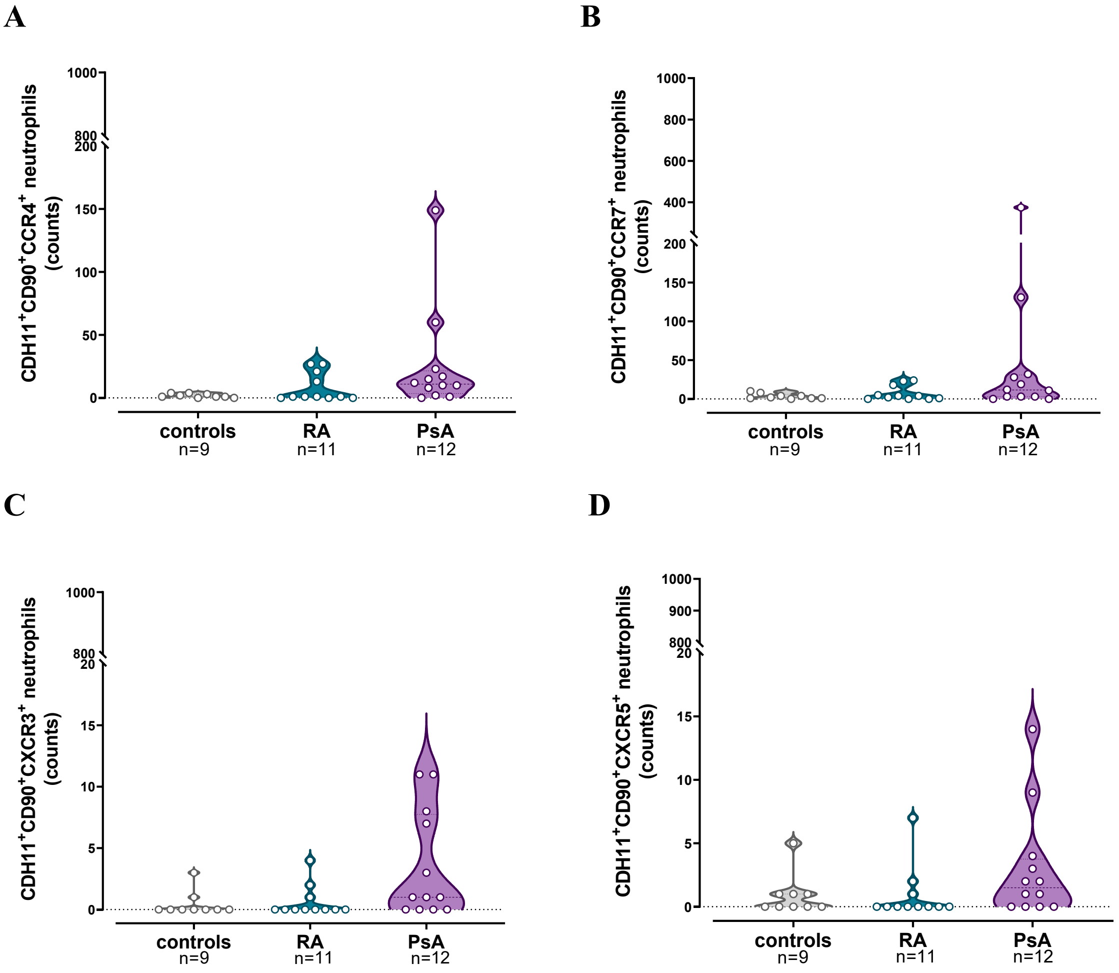

Supplementary Figure S25. $CD90^+CDH11^+$ neutrophils expressing CCR4, CCR7, CXCR3 or CXCR5. Violin plots showing the counts of (**A**) $CCR4^+$, (**B**) $CCR7^+$, (**C**) $CXCR3^+$, and (**D**) $CXCR5^+$ $CD90^+CDH11^+$ neutrophils, across the study groups. Each point corresponds to an individual (grey plot= controls, n= 9; cyan plot= RA, n= 11; purple plot= PsA, n= 12). Horizontal dotted lines represent the median (middle line) and the 25th (lower line) and 75th (upper line) percentiles. Groups were compared using Kruskal–Wallis test, followed by the two-stage step-up method of Benjamini, Krieger and Yekutieli for post-hoc analysis (HC vs RA vs PsA). Asterisks denote statistically significant differences between groups. (n: number of individuals; RA: rheumatoid arthritis; PsA: psoriatic arthritis; CDH11: cadherin-11; PDPN: podoplanin)

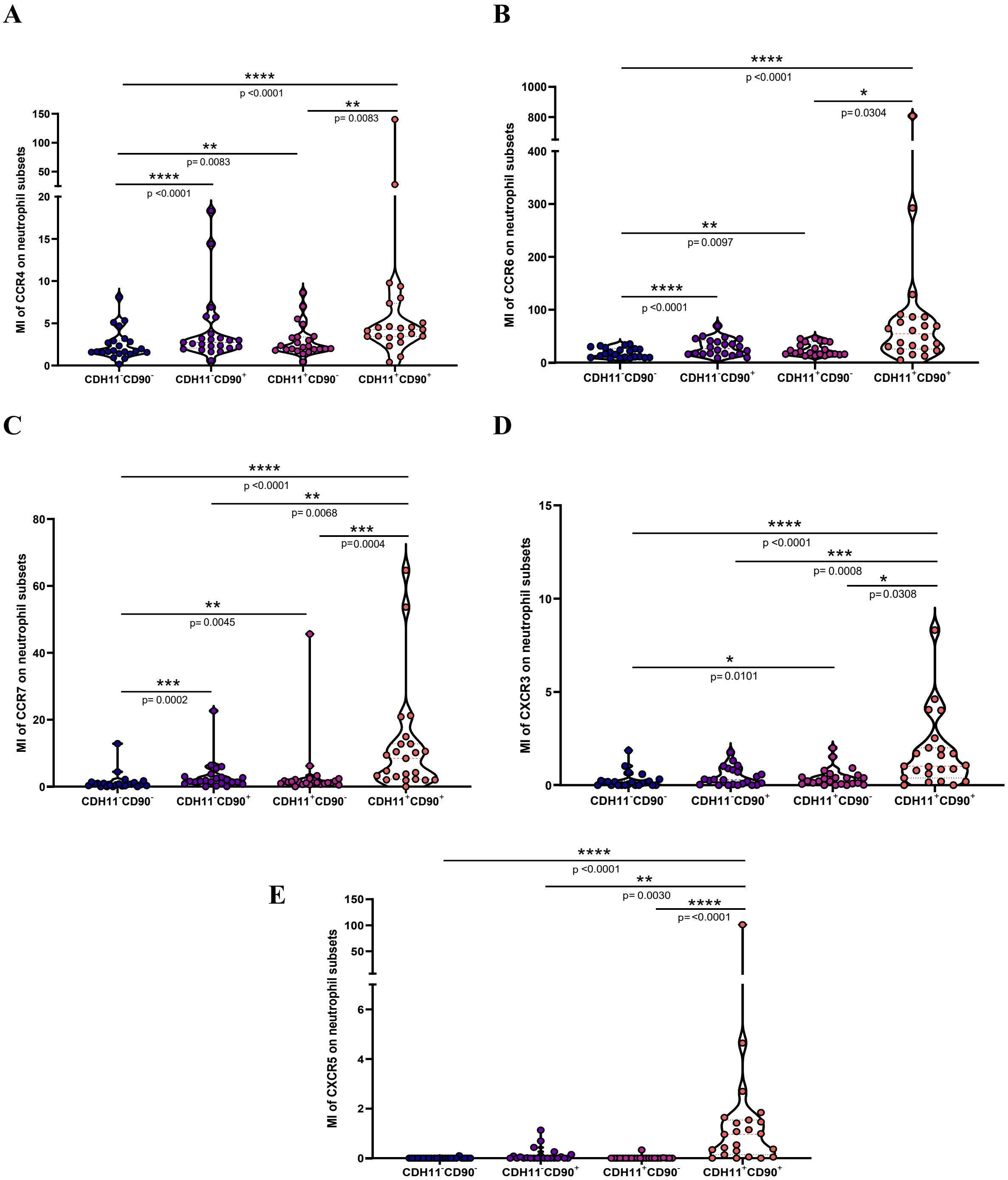

Supplementary Figure S26. Chemokine receptors are preferentially overexpressed on $CDH11^{+}CD90^{+}$ neutrophils in patients. Violin plots showing the median expression (MI) of (A) CCR4, (B) CCR6, (C) CCR7, (D) CXCR3, and (E) CXCR5 on $CDH11^{-}CD90^{-}$, $CDH11^{-}CD90^{+}$, cadherin-$11^{+}CD90^{-}$ and $CDH11^{+}CD90^{+}$ neutrophils, in patients with inflammatory arthritis (n= 23). Each point corresponds to an individual. Horizontal dotted lines represent the median (middle line) and the 25th (lower line) and 75th (upper line) percentiles. Groups were compared using Friedman test for paired measurements, followed by Dunn's correction for post-hoc analysis. (CDH11: cadherin-11; *, $p< 0.050$; **, $p\leq 0.010$; ***, $p\leq 0.001$; ****, $p\leq 0.0001$)

A

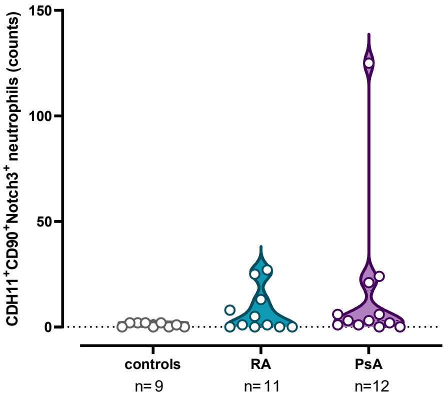

B

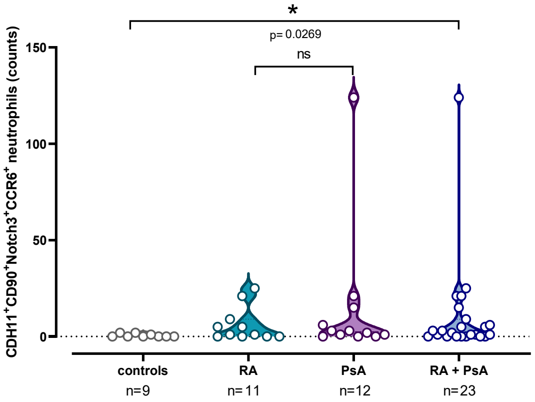

C

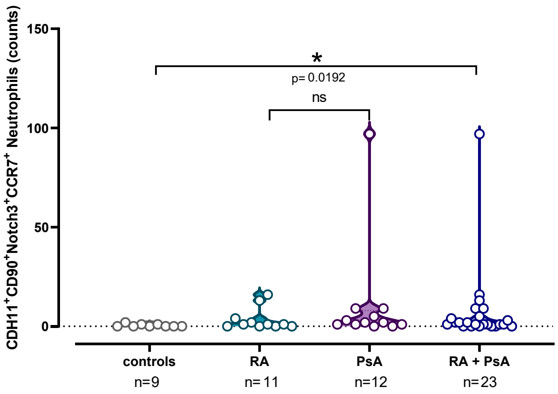

Supplementary Figure S27. $CCR6^{+}$ and $CCR7^{+}$ $CDH11^{+}CD90^{+}Notch3^{+}$ neutrophils are increased in patients. (**A**) Violin plots showing the counts of $CDH11^{+}CD90^{+}Notch3^{+}$ neutrophils, across the study groups. Each point corresponds to an individual. (**B**) Violin plots showing the counts of $CDH11^{+}CD90^{+}Notch3^{+}CCR6^{+}$ neutrophils in patient groups and controls. Each point corresponds to an individual. (**C**) Violin plots showing the counts of $CDH11^{+}CD90^{+}Notch3^{+}CCR7^{+}$ neutrophils in patient groups and controls. Each point corresponds to an individual (grey plot= controls, n= 9; cyan plot= RA, n= 11; purple plot= PsA, n= 12; blue plot= RA+PsA, n= 23). Horizontal dotted lines represent the median (middle line) and the 25th (lower line) and 75th (upper line) percentiles. Groups were compared using Kruskal–Wallis test, followed by the two-stage step-up method of Benjamini, Krieger and Yekutieli for post-hoc analysis (HC vs RA vs PsA) and Mann-Whitney U test (HC vs merged RA+PsA). Asterisks denote statistically significant differences between groups. (n: number of individuals; RA: rheumatoid arthritis; PsA: psoriatic arthritis; CDH11: cadherin-11; ns: not significant; *, $p< 0.050$)

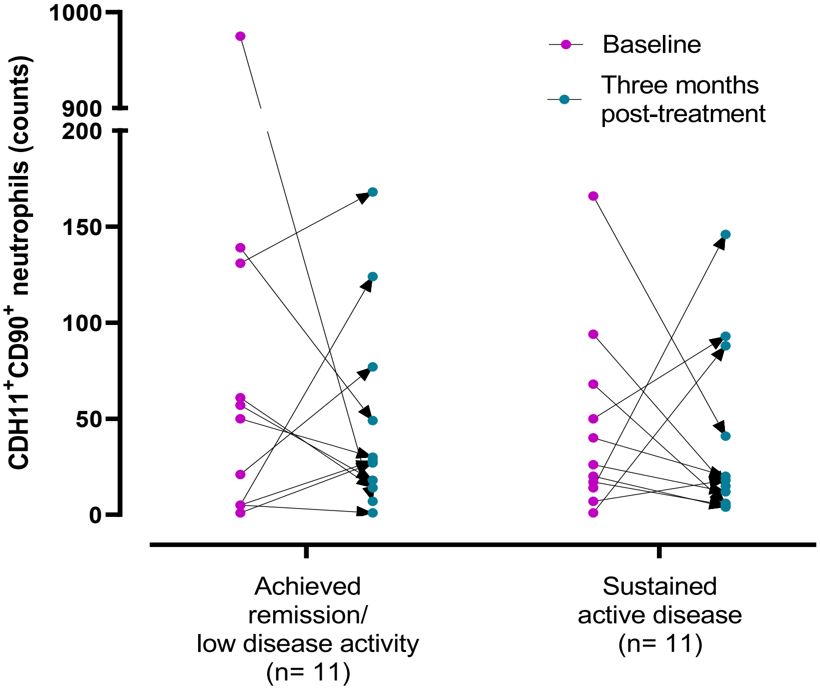

Supplementary Figure S28. CDH11$^+$CD90$^+$ neutrophil counts in re-evaluated patients three months post-treatment are comparable to baseline, despite changes in disease activity. CDH11$^+$CD90$^+$ neutrophil counts in patients who became inactive (n= 11) or remained active (n= 11) post-treatment, measured at baseline and in their three-month follow-up. Each point corresponds to an individual patient (magenta points= baseline levels; cyan points= three months post-treatment levels). Comparison of paired samples between timepoints was performed using Wilcoxon test. (CDH11: cadherin-11)

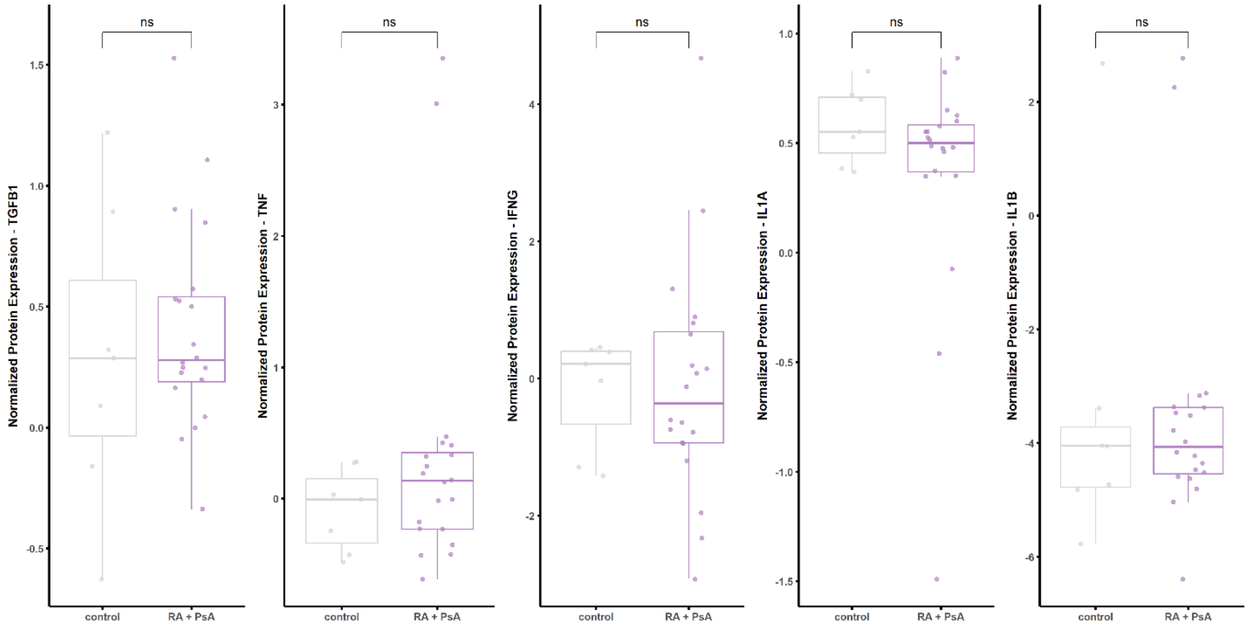

Supplementary Figure S29. Similar levels of IL-1A, IL-1B, TNFα, IFNγ and TGFβ between patients and controls. Boxplots showing protein levels in NPX values in patients and controls. Each point corresponds to an individual (grey plot= patients with detectable CDH11^{+} fibroblasts, n= 9; purple plot= patients without detectable CDH11^{+} fibroblasts, n= 11). Horizontal line represents the median, while the whiskers extend to the 25th and 75th percentiles. Groups were compared using t-test (RA: rheumatoid arthritis; PsA: psoriatic arthritis; TGFB1: transforming growth factor beta-1; TNF: tumor necrosis factor; IFNG: interferon-γ; IL-1: interleukin-1; ns: not significant)

1.2 Supplementary Tables

Supplementary Table S1. Demographics, clinical and laboratory data of individuals enrolled in the study and analyzed by mass cytometry

Characteristics	Controls (n=9)	RA (n=11)	PsA (n=12)
Age (years), median (range)	48 (38-60)	56 (33-65)	51 (19-57)
Gender (female), n (%)	5 (56%)	11 (100%)	9 (75%)
Smoking status (smoker), n (%)	4 (44%)	4 (36%)	2 (17%)
Treatment			
naïve, n (%)	N/A	4 (36%)	5 (42%)
csDMARDs-experienced, n (%)	N/A	5 (46%)	4 (33%)
b/tsDMARDs-experienced, n (%)	N/A	2 (18%)	3 (25%)
CRP (mg/l), mean (±SEM)	N/A	32.9 (± 23.7)	13.5 (± 5.1)
ESR (mm/h), mean (±SEM)	N/A	39 (± 8)	32 (± 8)
Disease activity score, mean (±SEM)	N/A	DAS28, 5.24 (± 0.29)	DAPSA, 19.65 (± 1.30)
RF, n (%)	N/A	9 (82%)	N/A
Anti-CCP, n (%)	N/A	7 (64%)	N/A
Axial involvement, n (%)	N/A	N/A	5 (42%)
Enthesitis, n (%)	N/A	N/A	1 (8%)
Dactylitis, n (%)	N/A	N/A	1 (8%)
BSA (≥ 3), n (%)	N/A	N/A	6 (50%)

RA: rheumatoid arthritis; PsA: psoriatic arthritis; csDMARDs: conventional synthetic disease modifying antirheumatic drugs; b/tsDMARDs: biologic/targeted-synthetic disease modifying antirheumatic drugs; CRP: C-reactive protein; ESR: erythrocyte sedimentation rate; DAS28: Disease Activity Score-28; RF: rheumatoid factor; DAPSA: Disease Activity Index for Psoriatic Arthritis; anti-CCP: anti-cyclic citrullinated peptide; BSA: body surface area

Supplementary Table S2. The antibody panel used for mass cytometry. All antibodies were purchased from Standard BioTools Inc., San Francisco, CA, USA.

Metal	Target	Clone
89Y	CD45	HI30
110Cd	CD34	581
141Pr	CD196/CCR6	G034E3
142Nd	PDPN	NC-08
143Nd	CD123/IL-3R	6H6
144Nd	CD19	HIB19
145Nd	CD4	RPA-T4
146Nd	CD8a	RPA-T8
147Sm	CD11c	Bu15
148Nd	CD16	3G8
149Sm	CD45RO	UCHL1
150Nd	CD45RA	HI100
151Eu	CD161	HP-3G10
152Sm	CD194/CCR4	L291H4
153Eu	CD25	BC96
154Sm	CD27	O323
155Gd	CD57	HCD57
156Gd	CD183/CXCR3	G025H7
158Gd	CD185/CXCR5	J252D4
159Tb	CD90/Thy-1	5E10
160Gd	CD28	CD28.2
161Dy	CD38	HB-7
163Dy	CD56/NCAM	NCAM16.2
164Dy	TCRgd	B1
165 Ho	Notch3	MHN3-21
166Er	CD294	BM16
167Er	CD197/CCR7	G043H7
168Er	CD14	63D3
169Tm	CDH11	16G5
170Er	CD3	UCHT1
171Yb	CD20	2H7
172Yb	CD66b	G10F5
173Yb	HLA-DR	LN3
174Yb	IgD	IA6-2
176Yb	CD127/IL-7Ra	A019D5

Supplementary Table S3. Fibroblast phenotypes based on all possible combinations of CDH11, CD34, Notch3 and CD90.

Annotation	Phenotype
FB1	$CDH11^{-}CD34^{-}Notch3^{-}CD90^{-}$
FB2	$CDH11^{+}CD34^{-}Notch3^{-}CD90^{-}$
FB3	$CDH11^{-}CD34^{+}Notch3^{-}CD90^{-}$
FB4	$CDH11^{-}CD34^{-}Notch3^{+}CD90^{-}$
FB5	$CDH11^{-}CD34^{-}Notch3^{-}CD90^{+}$
FB6	$CDH11^{+}CD34^{+}Notch3^{-}CD90^{-}$
FB7	$CDH11^{+}CD34^{-}Notch3^{+}CD90^{-}$
FB8	$CDH11^{+}CD34^{-}Notch3^{-}CD90^{+}$
FB9	$CDH11^{-}CD34^{+}Notch3^{+}CD90^{-}$
FB10	$CDH11^{-}CD34^{+}Notch3^{-}CD90^{+}$
FB11	$CDH11^{-}CD34^{-}Notch3^{+}CD90^{+}$
FB12	$CDH11^{+}CD34^{+}Notch3^{+}CD90^{-}$
FB13	$CDH11^{+}CD34^{+}Notch3^{-}CD90^{+}$
FB14	$CDH11^{+}CD34^{-}Notch3^{+}CD90^{+}$
FB15	$CDH11^{-}CD34^{+}Notch3^{+}CD90^{+}$
FB16	$CDH11^{+}CD34^{+}Notch3^{+}CD90^{+}$

CDH11: cadherin-11

Supplementary Table S4. Fibrocyte phenotypes based on all possible combinations of CDH11, PDPN, CD90 and Notch3

Annotation	Phenotype
FC1	$CDH11^{-}PDPN^{-}Notch3^{-}CD90^{-}$
FC2	$CDH11^{+}PDPN^{-}Notch3^{-}CD90^{-}$
FC3	$CDH11^{-}PDPN^{+}Notch3^{-}CD90^{-}$
FC4	$CDH11^{-}PDPN^{-}Notch3^{+}CD90^{-}$
FC5	$CDH11^{-}PDPN^{-}Notch3^{-}CD90^{+}$
FC6	$CDH11^{+}PDPN^{+}Notch3^{-}CD90^{-}$
FC7	$CDH11^{+}PDPN^{-}Notch3^{+}CD90^{-}$
FC8	$CDH11^{+}PDPN^{-}Notch3^{-}CD90^{+}$
FC9	$CDH11^{-}PDPN^{+}Notch3^{+}CD90^{-}$
FC10	$CDH11^{-}PDPN^{+}Notch3^{-}CD90^{+}$
FC11	$CDH11^{-}PDPN^{-}Notch3^{+}CD90^{+}$
FC12	$CDH11^{+}PDPN^{+}Notch3^{+}CD90^{-}$
FC13	$CDH11^{+}PDPN^{+}Notch3^{-}CD90^{+}$
FC14	$CDH11^{+}PDPN^{-}Notch3^{+}CD90^{+}$
FC15	$CDH11^{-}PDPN^{+}Notch3^{+}CD90^{+}$
FC16	$CDH11^{+}PDPN^{+}Notch3^{+}CD90^{+}$

CDH11: cadherin-11; PDPN: podoplanin

Supplementary Table S5. Phenotypic definition of the 12 identified leukocyte subpopulations

**Subpopulation**	**Phenotype**
Neutrophils	$CD45^{lo}CD66b^{+}CD294^{-}CD16^{+}$
Eosinophils	$CD45^{lo}CD66b^{+}CD294^{+}CD16^{-}$
Basophils	$CD45^{+}CD66b^{-}CD19^{-}CD20^{-}CD3^{-}CD56^{-}HLA\text{-}DR^{-}CD11c^{-}CD123^{+}CD294^{+}$
Monocytes	$CD45^{+}CD66b^{-}CD19^{-}CD20^{-}CD3^{-}CD56^{-}CD11c^{+}HLA\text{-}DR^{+}$
NK cells	$CD45^{+}CD66b^{-}CD19^{-}CD20^{-}CD3^{-}CD14^{-}CD45RA^{+}CD123^{-}CD56^{+}$
DC	$CD45^{+}CD66b^{-}CD19^{-}CD20^{-}CD3^{-}CD14^{-}HLA\text{-}DR^{+}$
ILC	$CD45^{+}CD3^{-}CD19^{-}CD56^{-}CD14^{-}CD16^{-}CD11c^{-}TCR\gamma\delta^{-}CD123^{-}CD127^{+}$
MAIT/iNKT	$CD45^{+}CD66b^{-}CD19^{-}CD20^{-}CD14^{-}CD11c^{-}CD3^{+}CD4^{-}CD28^{+}CD161^{hi}$
γδ T cells	$CD45^{+}CD66b^{-}CD19^{-}CD20^{-}CD14^{-}CD11c^{-}CD3^{+}CD4^{-}CD8^{-}TCR\gamma\delta^{+}$
$CD4^{+}$ T cells	$CD45^{+}CD66b^{-}CD19^{-}CD20^{-}CD14^{-}CD11c^{-}CD3^{+}TCR\gamma\delta^{-}CD4^{+}CD8^{-}$
$CD8^{+}$ T cells	$CD45^{+}CD66b^{-}CD19^{-}CD20^{-}CD14^{-}CD11c^{-}CD3^{+}TCR\gamma\delta^{-}CD4^{-}CD8^{+}CD161^{lo/-}$
B cells	$CD45^{+}CD66b^{-}CD56^{-}CD14^{-}CD19^{+}CD3^{-}$

NK: natural killer cells; DC: dendritic cells; MAIT: mucosal-associated invariant T cells; iNKT: invariant natural killer T cells; ILC: innate lymphoid cells

Supplementary Table S6. Clinical data and circulating fibroblast and fibrocyte counts and percentages obtained from 270 µl peripheral blood, for each individual enrolled in the study

	Baseline				Three months post-treatment				
	Circulating Fibroblasts		Circulating Fibrocytes		Disease activity	Circulating Fibroblasts		Circulating Fibrocytes	
	Counts (in 270 µl)	% in intact cells	Counts (in 270 µl)	% in intact cells		Counts (in 270 µl)	% in intact cells	Counts (in 270 µl)	% in intact cells
RA1	0	0.0000	6	0.0020	L	3	0.0011	45	0.0158
RA2	0	0.0000	8	0.0029	L	0	0.0000	4	0.0015
RA3	0	0.0000	86	0.0219	L	3	0.0007	268	0.0638
RA4	2	0.0003	8	0.0014	H	0	0.0000	29	0.0054
RA5	10	0.0021	8	0.0016	L	0	0.0000	12	0.0063
RA6	0	0.0000	21	0.0050	M	0	0.0000	4	0.0164
RA7	2	0.0004	14	0.0025	M	4	0.0011	181	0.0506
RA8	1	0.0002	12	0.0030	H	1	0.0002	15	0.0029
RA9	4	0.0007	41	0.0075	L	2	0.0004	93	0.0170
RA10	5	0.0012	7	0.0017	H	2	0.0006	25	0.0073
RA11	2	0.0005	42	0.0100	H	7	0.0019	251	0.0684
PsA1	1	0.0003	8	0.0024	L	0	0.0000	4	0.0007
PsA2	0	0.0000	21	0.0055	M	5	0.0014	76	0.0216
PsA3	7	0.0015	7	0.0015	L	0	0.0000	33	0.0015
PsA4	0	0.0000	18	0.0029	L	0	0.0000	149	0.0282
PsA5	2	0.0004	24	0.0045	H	0	0.0000	12	0.0022
PsA6	3	0.0010	89	0.0285	L	0	0.0000	120	0.0372
PsA7	4	0.0020	19	0.0093	M	8	0.0017	158	0.0330
PsA8	1	0.0002	55	0.0110	L	0	0.0000	73	0.0259
PsA9	2	0.0003	10	0.0016	L	2	0.0003	155	0.0254
PsA10	2	0.0004	20	0.0043	H	0	0.0000	54	0.0097
PsA11	1	0.0002	29	0.0066	H	0	0.0000	36	0.0069
PsA12	2	0.0003	22	0.0063	N/A	N/A	N/A	N/A	N/A
HC1	0	0.0000	2	0.0011	N/A	N/A	N/A	N/A	N/A
HC2	0	0.0000	11	0.0027	N/A	N/A	N/A	N/A	N/A
HC3	1	0.0004	7	0.0025	N/A	N/A	N/A	N/A	N/A
HC4	1	0.0003	30	0.0081	N/A	N/A	N/A	N/A	N/A
HC5	0	0.0000	4	0.0019	N/A	N/A	N/A	N/A	N/A
HC6	1	0.0003	52	0.0169	N/A	N/A	N/A	N/A	N/A
HC7	0	0.0000	4	0.0013	N/A	N/A	N/A	N/A	N/A
HC8	0	0.0000	7	0.0015	N/A	N/A	N/A	N/A	N/A
HC9	1	0.0003	21	0.0064	N/A	N/A	N/A	N/A	N/A

RA: rheumatoid arthritis; PsA: psoriatic arthritis; HC: healthy controls; CRP: C-reactive protein; ESR: erythrocyte sedimentation rate; for disease activity: L=low, M=moderate, H=high

Supplementary Table S7. Clinical and laboratory characteristics and circulating fibroblast and fibrocyte counts obtained from 270 µl peripheral blood for each patient enrolled in the study

Patient ID	Circulating fibroblast counts (in 270 µl)	Circulating fibrocyte counts (in 270 µl)	CRP (mg/l)	ESR (mm/h)	Disease activity score	RF	anti-CCP	Treatment status
RA1	0	6	41.3	60	6.52	1	1	csDMARDs-experienced
RA2	0	8	4.6	33	5.24	1	1	naïve
RA3	0	86	6.0	24	4.86	1	1	b/tsDMARDs-experienced
RA4	2	8	2.6	20	5.49	0	0	naïve
RA5	10	8	2.7	46	4.82	1	1	csDMARDs-experienced
RA6	0	21	6.4	25	5.36	1	1	csDMARDs-experienced
RA7	2	14	18.2	49	4.80	1	1	csDMARDs-experienced
RA8	1	12	3.5	35	4.40	1	0	csDMARDs-experienced
RA9	4	41	267.3	101	7.40	1	0	naïve
RA10	5	7	3.4	23	4.60	1	1	b/tsDMARDs-experienced
RA11	2	42	5.8	8	4.20	0	0	naïve
PsA1	1	8	30.8	61	22.10	N/A	N/A	naïve
PsA2	0	21	2.7	15	19.74	N/A	N/A	b/tsDMARDs-experienced
PsA3	7	7	8.7	16	15.00	N/A	N/A	csDMARDs-experienced
PsA4	0	18	12.6	34	25.00	N/A	N/A	b/tsDMARDs-experienced
PsA5	2	24	3.0	16	25.00	N/A	N/A	b/tsDMARDs-experienced
PsA6	3	89	0.9	7	15.00	N/A	N/A	naïve
PsA7	4	19	0.6	21	16.00	N/A	N/A	csDMARDs-experienced
PsA8	1	55	7.0	6	16.00	N/A	N/A	csDMARDs-experienced
PsA9	2	10	10.0	25	15.00	N/A	N/A	naïve
PsA10	2	20	15.0	51	24.00	N/A	N/A	naïve
PsA11	1	29	10.2	37	18.00	N/A	N/A	naïve
PsA12	2	22	60.0	90	25.00	N/A	N/A	csDMARDs-experienced

RA: rheumatoid arthritis; PsA: psoriatic arthritis; CRP: C-reactive protein; ESR: erythrocyte sedimentation rate; RF: rheumatoid factor; anti-CCP: anti-cyclic citrullinated peptide; csDMARDs: conventional synthetic disease modifying antirheumatic drugs; b/tsDMARDs: biologic/targeted-synthetic disease modifying antirheumatic drugs

Disease activity score corresponds to Disease Activity Score-28 (DAS28) for RA patients and to Disease Activity Index for Psoriatic Arthritis (DAPSA) for PsA patients

For RF and anti-CCP: 0=negative, 1=positive for the respective auto-antibodies

Supplementary Table S8. Correlations of the numbers of circulating CDH11$^+$ fibroblasts and CDH11$^+$ fibrocytes with CDH11$^+$CD90$^+$ leukocyte subpopulations in patients. Correlation coefficients between the variables were calculated according to Spearman's rank correlation coefficient (r_s).

**CDH11$^+$CD90$^+$ leukocyte subpopulations**	**CDH11$^+$ fibroblasts**		**CDH11$^+$ fibrocytes**	
	r_s	p value	r_s	p value
Neutrophils	0.341	0.111	**0.498**	**0.015**
Eosinophils	-0.006	0.978	-0.159	0.469
Basophils	-0.088	0.690	0.162	0.461
Monocytes	0.203	0.353	**0.698**	**0.001**
NK cells	0.180	0.410	**0.548**	**0.007**
DC	-0.026	0.908	**0.765**	**0.001**
ILC	0.338	0.115	**0.492**	**0.017**
MAIT/iNKT	0.234	0.283	0.268	0.216
γδ T cells	0.026	0.905	**0.607**	**0.014**
CD4$^+$ T cells	0.150	0.495	0.407	0.054
CD8$^+$ T cells	0.382	0.072	0.238	0.275
B cells	0.268	0.217	0.112	0.611

CDH11: cadherin-11; NK: natural killer cells; DC: dendritic cells; MAIT: mucosal-associated invariant T cells; iNKT: invariant natural killer T cells; ILC: innate lymphoid cells

Supplementary Table S9. Statistically significant correlations of plasma protein levels and circulating CDH11$^+$ fibrocytes, CCR7$^+$ fibrocytes and CDH11$^+$CD90$^+$CCR6$^+$ neutrophils in patients. Correlation coefficients between the variables were calculated according to Pearson's correlation coefficient (r).

**Circulating cell populations**	**Plasma protein**	**r**	**p value**	**p_{adj}**
**CDH11$^+$ fibrocytes**	IL-33	0.747	0.0002	0.1
**CCR7$^+$ fibrocytes**	IL-33	0.779	0.0001	0.1
**CDH11$^+$CD90$^+$CCR6$^+$ neutrophils**	CCL28	0.743	0.0003	0.1
**CDH11$^+$CD90$^+$CCR6$^+$ neutrophils**	DBNL	0.740	0.0003	0.1
**CDH11$^+$CD90$^+$CCR6$^+$ neutrophils**	NCK2	0.758	0.0002	0.1
**CDH11$^+$CD90$^+$CCR6$^+$ neutrophils**	BCR	0.738	0.0003	0.1

CDH11: cadherin-11; DBNL: Drebrin-like protein; BCR: breakpoint cluster region protein
